# Graph informed biomarker discovery framework using transcriptomic machine learning for glioblastoma prognosis

**DOI:** 10.1038/s41598-026-58062-4

**Published:** 2026-06-23

**Authors:** Osama Mahmoud, Mahmoud Mounir, Walaa Gad

**Affiliations:** Information Systems Department, Faculty of Computer and Information Sciences, Ain Shams University, Cairo, Egypt

**Keywords:** Glioblastoma, Transcriptomics, Prognostic modeling, Graph-informed machine learning, Weighted protein–protein interaction, External validation, Biomarkers, Cancer, Computational biology and bioinformatics, Oncology

## Abstract

Identifying reproducible, interpretable prognostic signals from high-dimensional transcriptomics remains challenging because gene-level models often ignore network context. We developed Graph-Informed Biomarker Discovery (GIBD), a locked transcriptomics-only framework for primary glioblastoma that integrates RNA-seq expression with high-confidence STRING topology through weighted protein–protein interaction (WPPI) self-preserving feature construction. Model development, feature selection, scaler fitting, threshold selection, and locking used The Cancer Genome Atlas (TCGA) only, followed by post-lock external validation in the Chinese Glioma Genome Atlas (CGGA). The final TCGA cohort included 147 patients, and the empirical TCGA median overall survival of 357 days defined binary risk groups. The binary-evaluable CGGA cohort included 131 patients. The locked GIBD-XGBoost K100 model used 100 features (65 WPPI-derived, 35 raw-expression features) and threshold 0.53. TCGA out-of-fold AUC was 0.617. Post-lock CGGA validation yielded an AUC of 0.609, sensitivity of 73.9%, specificity of 50.6%, balanced accuracy of 62.3%, and a C-index of 0.537. SHAP identified *TSPAN13* as the strongest global contributor, and full-transcriptome TCGA GSEA identified 156 terms at FDR < 0.05, with coherent high-risk inflammatory, hypoxic, metabolic, extracellular-matrix, complement/coagulation, and angiogenic enrichment. GIBD preserved an external transcriptomic risk-prioritization signal requiring prospective recalibration and multimodal validation before translational use.

**Supplementary Information:**

The online version contains supplementary material available at 10.1038/s41598-026-58062-4.

## Introduction

Glioblastoma (GBM) remains one of the most aggressive adult brain tumors, with poor survival despite maximal safe resection, radiotherapy, and temozolomide-based chemotherapy^1,2^. Its clinical course is shaped by diffuse infiltration, recurrence, treatment resistance, and substantial interpatient and intratumoral heterogeneity^3–6^. These features limit the ability of conventional clinicopathologic variables alone to capture the molecular diversity underlying prognosis and motivate reproducible molecular risk-stratification frameworks that remain interpretable and externally evaluable.

RNA sequencing (RNA-seq) provides a broad view of tumor transcriptional state and is widely used in computational oncology^7^. However, transcriptomic prognosis modeling is vulnerable to overfitting because the number of measured genes often exceeds the number of clinically annotated samples^8^. Conventional gene-signature models, including regularized linear approaches, can be interpretable but may underrepresent nonlinear dependencies^9^. More complex omics and network-informed studies across cancer types have emphasized high-dimensional risk scoring and biomarker discovery, supporting structured prognostic representations that incorporate biological context^10–12^.

Protein–protein interaction (PPI) networks provide one way to incorporate such context^13,14^. They encode prior knowledge about functional relationships among gene products and can guide feature construction in high-dimensional transcriptomic models^13–15^. However, a static PPI network does not measure patient-specific protein abundance or interaction activity. In this study, STRING topology was therefore used only as an external graph prior for contextualizing measured RNA-seq signals, not as patient-specific proteomic evidence.

Motivated by these considerations, Graph-Informed Biomarker Discovery (GIBD) was implemented as a locked, transcriptomics-only, graph-informed risk-prioritization workflow for glioblastoma prognosis. The framework integrates RNA-seq expression, high-confidence STRING topology^15^, weighted protein–protein interaction (WPPI) self-preserving feature construction, The Cancer Genome Atlas (TCGA)-only model development, and post-lock external validation in the Chinese Glioma Genome Atlas (CGGA). Detailed WPPI implementation, including neighbor eligibility, weighting, and self-preserving mixtures, is described in the Methods and Supplementary Information.

A central objective was leakage-controlled reproducibility. TCGA was used for cohort curation, endpoint definition, WPPI feature construction, feature selection, candidate model selection, threshold selection, and final model locking, whereas CGGA was reserved as a post-lock external validation cohort^16,17^. CGGA was not used to guide feature construction, feature selection, model selection, hyperparameter selection, threshold selection, pathway ranking, or scaling-parameter fitting.

Post-lock interpretability and pathway analyses were used only for contextualization. SHAP and LIME were applied after model locking to explain the frozen model at global and local levels^18,19^. Full-transcriptome TCGA preranked GSEA was treated as the primary pathway-contextualization analysis^20,21^, whereas locked-signature ORA and Enrichr-style analyses using curated pathway resources, including Reactome and Enrichr, were treated as supplementary cross-checks^22,23^. These analyses were not used for feature selection, model tuning, threshold selection, or clinical validation.

**Fig. 1:**
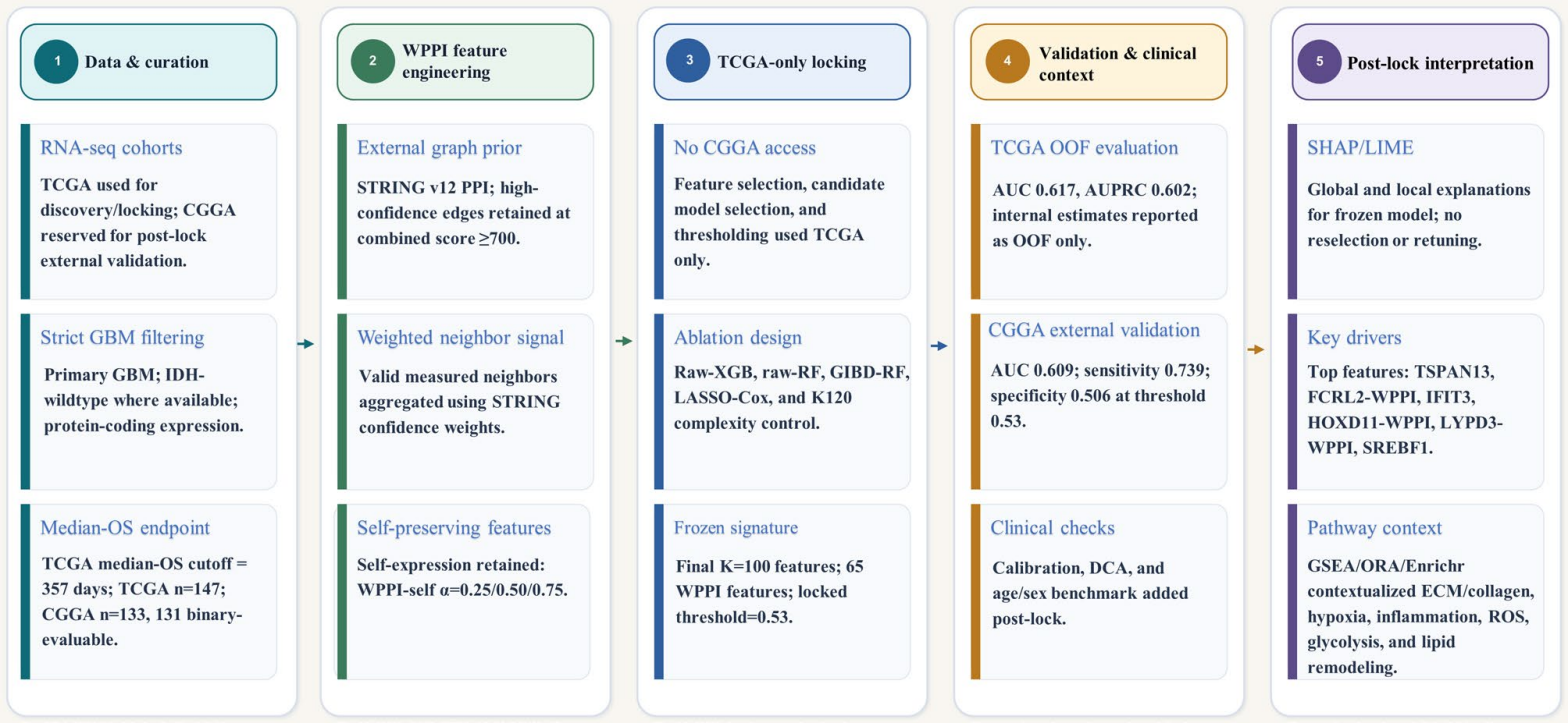
GIBD study workflow. Overview of the locked GIBD workflow for primary glioblastoma prognosis. TCGA RNA-seq and survival data were used for cohort curation, median-OS endpoint definition, preprocessing, WPPI feature construction, TCGA-only feature selection, model development, threshold selection, and final model locking. The CGGA was introduced only after the feature set, scaler, classifier, and operating threshold were frozen, and was used for post-lock external validation only. Post-lock analyses included ablation, calibration and decision curve analysis, clinical-covariate benchmarking, SHAP/LIME explanation, full-transcriptome GSEA, locked-signature ORA/Enrichr-style cross-checks, and PPI threshold sensitivity auditing. All TCGA-based development decisions were completed before the CGGA was introduced for post-lock external validation.

Finally, glioblastoma heterogeneity extends beyond transcriptomics. Imaging-texture, shape-and-intensity, MR-spectroscopy, metabolic-imaging, and spatial response studies demonstrate that GBM heterogeneity is multimodal and regionally variable^24–28^. Accordingly, GIBD should be interpreted as a transcriptomic risk-prioritization layer rather than a replacement for imaging, histopathology, molecular testing, or multidisciplinary clinical assessment. Table 1 provides concise methodological context, and Fig. 1 summarizes the locked workflow used to complete TCGA-based development before post-lock CGGA validation.

**Table 1:** Methodological context for GIBD relative to existing computational oncology approaches.

Area	Common use in computational oncology	Main limitation for this study context	GIBD positioning
Transcriptomic prognostic modeling	Uses gene-expression profiles to predict survival or risk groups	Often treats genes as independent predictors with limited network context	Adds graph-informed WPPI features while retaining transcriptomics-only inputs
Penalized and tree-based machine learning	Supports high-dimensional feature selection and nonlinear modeling	Can be difficult to interpret biologically if used without structured context	Combines TCGA-only feature selection, XGBoost modeling, ablation, and external validation
PPI/network-informed modeling	Incorporates prior biological relationships between genes or proteins	Network information can be overinterpreted as patient-specific biology	Uses STRING only as an external topology prior for feature construction
Multimodal GBM heterogeneity studies	Demonstrate imaging, metabolic, spatial, and molecular heterogeneity	Multimodal data are not consistently available across public cohorts	Focuses on a reproducible transcriptomics-only framework and discusses multimodal extension as future work
Explainable machine learning	Provides global and local interpretation of fitted models	Attribution methods do not establish causality or biomarker validation	Uses SHAP and LIME after model locking for model-level explanation

This table summarizes the methodological context for GIBD relative to existing computational oncology approaches. It is intended as a concise positioning aid rather than a direct performance comparison, because the cited approaches differ in cohorts, endpoints, modalities, validation designs, and prediction targets.

## Results

### Cohort composition and median-OS risk-label definition

The final analysis was based on a curated TCGA development cohort and an independent CGGA external-validation cohort. TCGA was used for endpoint definition, model development, out-of-fold evaluation, feature selection, candidate model selection, and final locking, whereas the CGGA was reserved for post-lock external validation only.

The empirical median overall survival in the curated TCGA cohort was 357 days. This cutoff was used to define the supervised median-OS risk label: patients with overall survival ≤ 357 days were assigned to the high-risk group, whereas patients with overall survival > 357 days were assigned to the low-risk group. The final TCGA development cohort included 147 patients, comprising 74 high-risk patients and 73 low-risk patients.

The same TCGA-derived 357-day cutoff was applied to the CGGA cohort after model locking. To avoid assigning artificial binary labels to censor-ambiguous samples, CGGA patients censored before 357 days were excluded from binary AUC and locked-threshold classification analyses. The final binary-evaluable CGGA external validation set included 131 patients, including 46 high-risk patients and 85 low-risk patients. The cohort roles, label definitions, and binary-evaluation counts are summarized in Table 2.

**Table 2:** Cohort and endpoint composition.

Cohort	Role in workflow	Data type	Patients used	Median-OS risk-label rule	Low-risk cases	High-risk cases	Binary-evaluation handling	Use in analysis
TCGA	Development, out-of-fold evaluation, model selection, and final locking	RNA-seq + overall survival	147	Empirical TCGA median OS = 357 days; high risk if OS < = 357 days; low risk if OS > 357 days	73	74	All included TCGA cases were binary-labeled using the TCGA median-OS rule	Cohort curation, endpoint definition, WPPI feature construction, fold-contained feature selection, model selection, threshold selection, final locking, and post-lock TCGA pathway contextualization
CGGA	Post-lock external validation	RNA-seq + overall survival	131 binary-evaluable patients from 133 eligible cases	Same TCGA-derived 357-day cutoff applied after model locking	85	46	Patients censored before 357 days were considered binary-ambiguous and excluded from binary AUC and locked-threshold classification metrics	Post-lock external evaluation only after freezing the feature set, scaler, classifier, and threshold; CGGA labels were used only to compute validation metrics

TCGA was used for cohort curation, endpoint definition, WPPI feature construction, fold-contained feature selection, model selection, threshold selection, and final model locking. TCGA expression was also used after locking for pathway contextualization. The CGGA was reserved for post-lock external validation. The binary prognostic endpoint was defined using the empirical TCGA median overall survival of 357 days. CGGA patients censored before 357 days were excluded from binary classification and AUC analyses to avoid assigning artificial risk labels to censor-ambiguous cases. OS, overall survival; WPPI, weighted protein–protein interaction; TCGA, The Cancer Genome Atlas; CGGA, Chinese Glioma Genome Atlas.

### Final WPPI-informed feature space and locked model configuration

**Fig. 2:**
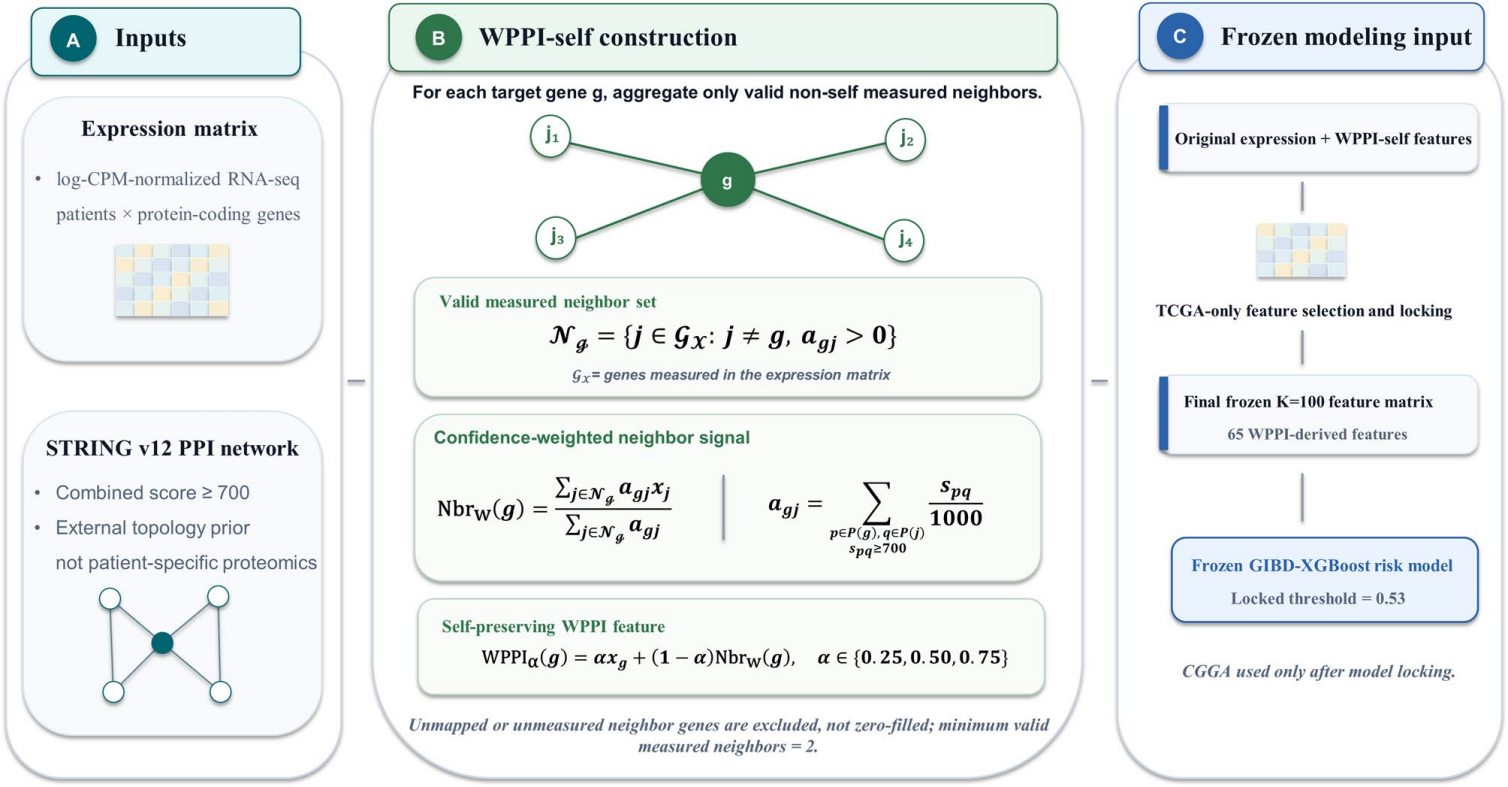
WPPI-self graph-informed feature construction. WPPI-self feature construction integrated measured RNA-seq expression with STRING v12 topology as an external graph prior. STRING edges were retained using a high-confidence combined-score cutoff of 700 or higher. For each target gene, valid measured non-self neighbors were identified, unmapped or unmeasured neighbors were excluded rather than zero-filled, and features were generated only when at least two measured non-self neighbors were available. Neighbor expression was aggregated via STRING confidence-weighted gene-level weights and combined with target-gene expression via pre-specified self-preserving alpha values of 0.25, 0.50, and 0.75. STRING was not interpreted as patient-specific protein abundance or patient-specific interaction activity.

The final model used a locked K = 100 feature space generated under TCGA-only development workflow. Of these 100 locked features, 65 were WPPI-derived features and 35 were raw-expression features, indicating that graph-informed representations were materially represented in the locked feature set while direct transcript-level measurements were retained. The final classifier was a frozen GIBD-XGBoost K100 model. The locked classification threshold was 0.53, which was selected from TCGA out-of-fold predictions via the pre-specified recall80_spec25 rule. This threshold and the final feature set were frozen before any CGGA external evaluation. The WPPI construction schematic is shown in Fig. 2, and the full algorithmic details are provided in Supplementary Table S1.

### TCGA-only locking, ablation, and post-lock CGGA validation

Internal model assessment was performed via TCGA out-of-fold predictions generated during the TCGA-only development workflow. The final locked GIBD-XGBoost K100 model achieved a TCGA OOF AUC of 0.617. At the locked threshold of 0.53, the TCGA OOF classification profile had a sensitivity of 82.4%, a specificity of 32.9%, and a balanced accuracy of 57.7%.

Ablation analyses were then used to compare the locked graph-informed model against raw-expression and model-family comparators. These comparisons included raw-expression XGBoost K100, raw-expression random forest K100, graph-informed GIBD-RF K100, raw-expression LASSO-Cox K100, and a GIBD-XGBoost K120 complexity-control model. All non-champion comparators were selected on the basis of TCGA-only out-of-fold performance and then evaluated on the same post-lock CGGA cohort.

In the CGGA external validation, the final GIBD-XGBoost K100 model achieved an AUC of 0.609, a sensitivity of 73.9%, a specificity of 50.6%, a balanced accuracy of 62.3%, and a C-index of 0.537. Among the main K100 comparators, raw-expression XGBoost had the same TCGA OOF AUC of 0.617 but lower CGGA AUC and balanced accuracy than GIBD-XGBoost K100. Compared with the raw-RF model, the raw-expression random forest model showed increased sensitivity but low specificity, whereas the GIBD-RF model improved specificity relative to raw-RF but did not match the balanced external profile of the GIBD-XGBoost model. Raw LASSO-Cox performed the weakest externally in this locked binary median-OS evaluation setting, highlighting the difference between a regularized survival-oriented linear comparator and the final nonlinear binary classifier.

This pattern indicates that the comparator models favored different operating trade-offs rather than uniformly outperforming the locked model. Raw-RF achieved higher sensitivity by assigning more samples to the high-risk class, but this came with substantially reduced specificity. In contrast, GIBD-RF improved specificity but reduced high-risk sensitivity. The final GIBD-XGBoost K100 model was therefore retained not because it dominated every individual metric, but because it provided a compact, graph-informed, sensitivity-prioritized external profile with a higher balanced accuracy than the main K100 comparators at the locked threshold.

**Fig. 3:**
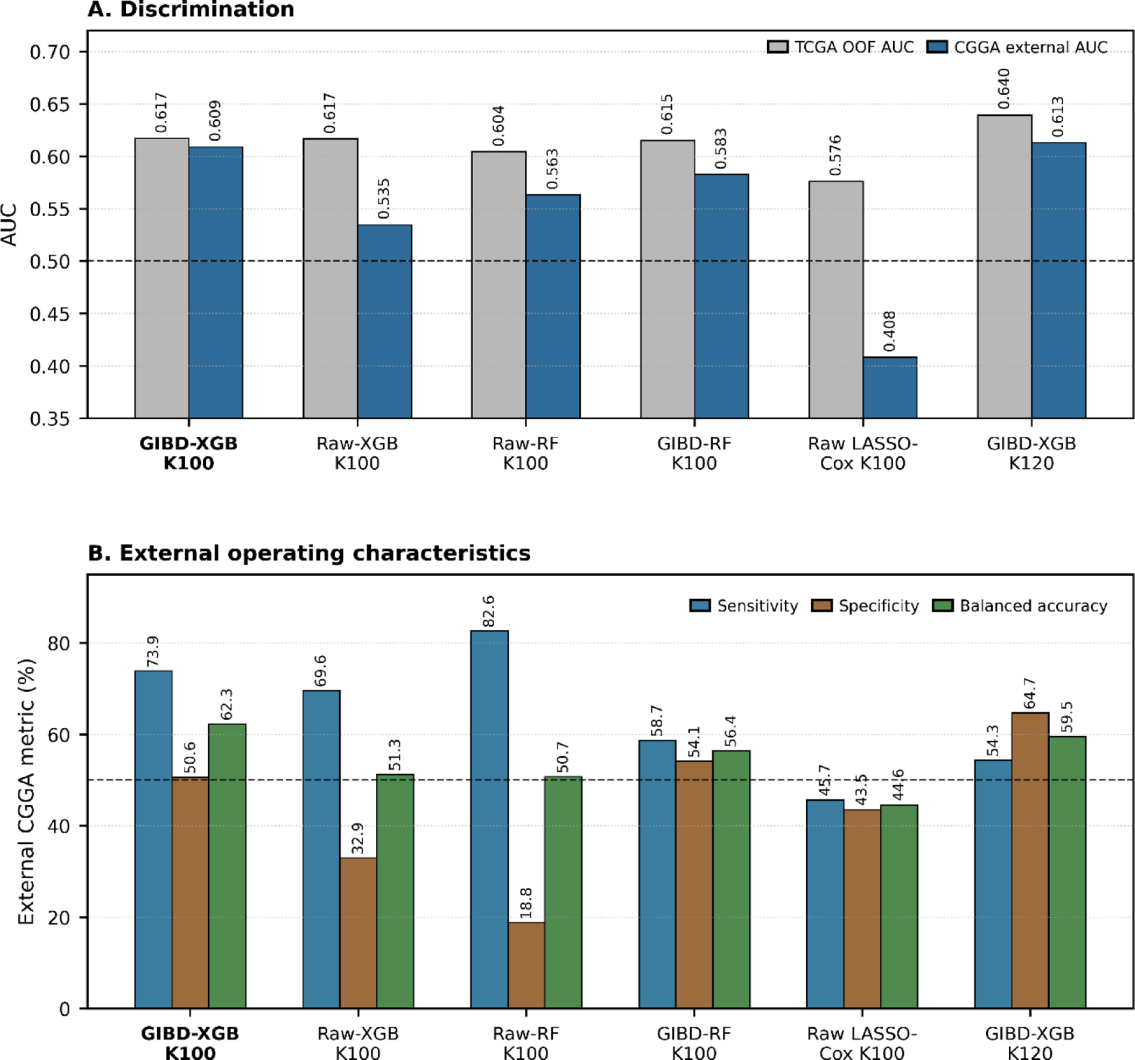
Ablation and external validation. Ablation and external validation profile of the final locked GIBD-XGBoost K100 model and comparator models. Panel A shows the TCGA out-of-fold and CGGA external AUCs. Panel B shows CGGA sensitivity, specificity, and balanced accuracy at model-specific TCGA-derived operating thresholds. The final GIBD-XGBoost K100 model uses 100 features, including 65 WPPI-derived and 35 raw-expression features, with a locked threshold of 0.53. In CGGA, the final GIBD-XGBoost K100 model achieved an AUC of 0.609, sensitivity of 73.9%, specificity of 50.6%, balanced accuracy of 62.3%, and a C-index of 0.537. Comparator models were selected using TCGA-only out-of-fold performance and evaluated in CGGA post-lock. Ablation patterns are interpreted descriptively, not as statistically significant pairwise superiority.

The K120 GIBD-XGBoost complexity-control model had a slightly higher CGGA AUC than did K100 but a lower sensitivity and balanced accuracy when a larger feature set was used. Therefore, K100 GIBD-XGBoost was retained as the final locked model because it provided a more compact and sensitivity-prioritized external operating profile. Figure 3 summarizes the external operating profile of the final model and comparators, while Table 3 reports the corresponding exact TCGA OOF and CGGA external metrics.

**Table 3:** Ablation and external validation metrics.

Model	Feature source	Feature budget	TCGA-derived operating threshold	TCGA OOF AUC	CGGA AUC	CGGA sensitivity (%)	CGGA specificity (%)	CGGA balanced accuracy (%)	CGGA MCC	CGGA C-index	Role in analysis
GIBD-XGBoost	Raw + WPPI	K = 100	0.530	0.617	0.609	73.9	50.6	62.3	0.237	0.537	Final locked model
Raw-XGBoost	Raw expression only	K = 100	0.565	0.617	0.535	69.6	32.9	51.3	0.026	0.530	Raw nonlinear comparator
Raw-Random Forest	Raw expression only	K = 100	0.365	0.604	0.563	82.6	18.8	50.7	0.018	0.515	Raw tree-ensemble comparator
GIBD-Random Forest	Raw + WPPI	K = 100	0.375	0.615	0.583	58.7	54.1	56.4	0.122	0.502	Graph-informed model-family comparator
Raw LASSO-Cox	Raw expression only	K = 100	0.395	0.576	0.408	45.7	43.5	44.6	-0.107	0.475	Regularized survival-oriented linear comparator
GIBD-XGBoost	Raw + WPPI	K = 120	0.510	0.640	0.613	54.3	64.7	59.5	0.187	0.531	Graph-informed complexity-control comparator

Each model was developed via TCGA-only procedures and evaluated via CGGA only after model-specific features, parameters, and operating thresholds had been fixed. Operating thresholds were selected from TCGA out-of-fold predictions via the same pre-specified thresholding rule and were not adjusted via CGGA labels. The final K = 100 GIBD-XGBoost model was retained because it provided a compact, sensitivity-prioritized external operating profile. Metrics are reported descriptively; no formal pairwise statistical superiority testing was performed. OOF, out-of-fold; AUC, area under the receiver operating characteristic curve; MCC, Matthews correlation coefficient; C-index, concordance index; WPPI, weighted protein–protein interaction.

### Calibration, decision curve analysis, and clinical-covariate benchmarking

Calibration and decision curve analyses were added as post-lock clinical context assessments. The final GIBD score had a TCGA OOF AUC of 0.617, an AUPRC of 0.602, a Brier score of 0.258, an expected calibration error of 0.142, and a balanced accuracy of 57.7%. In the CGGA external cohort, the same locked score had an AUC of 0.609, an AUPRC of 0.434, a Brier score of 0.268, an expected calibration error of 0.208, and a balanced accuracy of 62.3%.

These calibration metrics indicated that the locked model should not be interpreted as a fully calibrated clinical probability model. In both the TCGA OOF and CGGA external evaluations, the Brier score was worse than the corresponding prevalence-null Brier score, supporting cautious interpretation of the predicted probabilities. Decision curve analysis revealed favorable internal net benefit relative to the treat-all and treat-none strategies at the locked threshold in the TCGA OOF cohort. In CGGA, the model remained favorable relative to treat-all at the locked threshold but not relative to treat-none, with the most favorable external decision curve range occurring at lower threshold probabilities.

Clinical-covariate benchmarking further contextualized the transcriptomic score. In the CGGA, the age-only and age-plus-sex logistic models had AUC values of 0.615 and 0.626, respectively, but had very low high-risk sensitivity at the locked operating point. Specifically, age-only achieved a balanced accuracy of 52.5%, sensitivity of 10.9%, and specificity of 94.1%, whereas age plus sex achieved a balanced accuracy of 53.6%, sensitivity of 13.0%, and specificity of 94.1%. An exploratory GIBD-score-plus-age-plus-sex model achieved an AUC of 0.660, a balanced accuracy of 63.8%, a sensitivity of 50.0%, and a specificity of 77.6%. This combined model was treated as contextual only and did not alter the final locked transcriptomic classifier.

Overall, these analyses support framing GIBD as a transcriptomics-based risk-prioritization signal rather than as a standalone clinical decision tool. Calibration, decision curve, and clinical-covariate benchmark visualizations are provided in Supplementary Figures S1–S3.

### Post-lock model explainability: global SHAP and external LIME

Post-lock SHAP analysis was performed on the frozen GIBD-XGBoost K100 model using TCGA samples. The analysis was used only to interpret the learned model structure and was not used for feature selection, threshold selection, model tuning, or pathway testing.

**Fig. 4:**
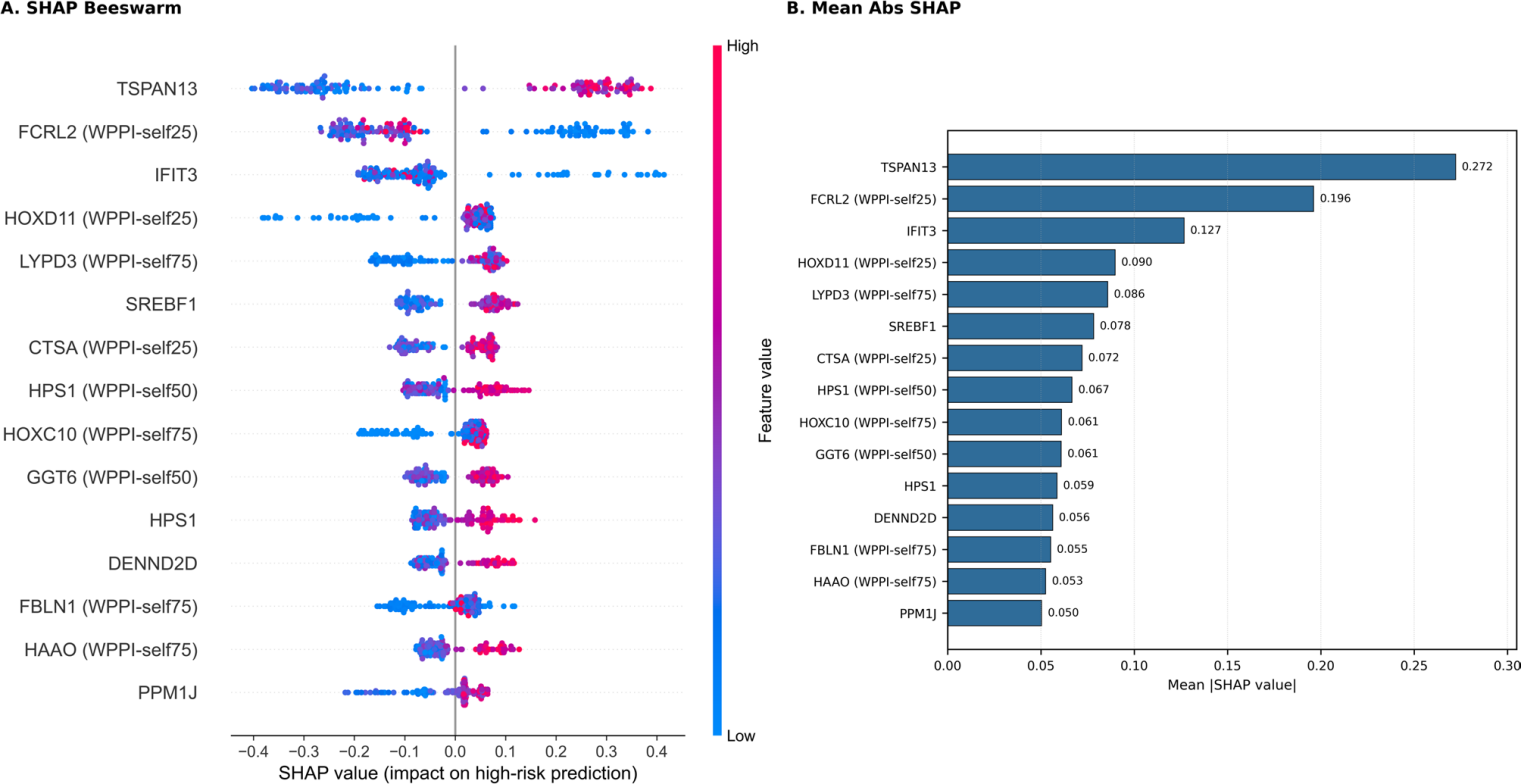
SHAP interpretation of the locked model. Post-lock global SHAP interpretation of the frozen GIBD-XGBoost K100 model using TCGA samples. Panel A shows the SHAP beeswarm plot for the top global contributors, and Panel B shows the corresponding mean absolute SHAP ranking. The strongest contributor was *TSPAN13*. The next highest-ranked contributors were *FCRL2* (WPPI-self25), *IFIT3*, *HOXD11* (WPPI-self25), *LYPD3* (WPPI-self75), *SREBF1*, *CTSA* (WPPI-self25), *HPS1* (WPPI-self50), *HOXC10* (WPPI-self75), and *GGT6* (WPPI-self50), with the full top-15 ranking shown in Panel B. WPPI-derived features comprised 9 of the top 15 contributors. SHAP was performed after model locking and represents model-level attribution, not causal biology or biomarker validation.

The strongest global SHAP contributor was *TSPAN13*. The next highest-ranked contributors were *FCRL2* (WPPI-self25), *IFIT3*, *HOXD11* (WPPI-self25), *LYPD3* (WPPI-self75), *SREBF1*, *CTSA* (WPPI-self25), *HPS1* (WPPI-self50), *HOXC10* (WPPI-self75), and *GGT6* (WPPI-self50), with the full top-15 ranking shown in Fig. 4B. Nine of the top 15 feature-level contributors were WPPI-derived features, supporting the contribution of graph-informed representations to the learned decision structure. Figure 4A shows the SHAP beeswarm plot for the top global contributors, and Fig. 4B summarizes the corresponding mean absolute SHAP ranking.

Exploratory SHAP interaction analyses were retained as supplementary model-level nonlinear dependency analyses. The highest-ranked interaction patterns included *TSPAN13*-*PPM1J*, *TSPAN13*-*IFIT3*, *HPS1* (WPPI-self50)-*FCRL2* (WPPI-self25), *TSPAN13*-*HPS1*, and *GGT6* (WPPI-self50)-*IFIT3*. These interaction values were interpreted as nonlinear model dependencies rather than biochemical interactions or evidence of molecular mechanisms.

LIME was used for post-lock local explanations of representative CGGA external cases. The selected examples included true positive, false positive, true negative, and false negative cases from the frozen external predictions. The false-positive and false-negative examples were close to the locked threshold, which is consistent with local decision-boundary uncertainty. LIME results are therefore reported as case-level model explanations and not as biological expression cutoffs or biomarker validation. External LIME examples are shown in Supplementary Figure S4. SHAP interaction and targeted dependence analyses are provided in Supplementary Figures S5 and S6.

### Full-transcriptome pathway contextualization by TCGA GSEA

Full-transcriptome preranked GSEA was performed after model locking to contextualize the TCGA median-OS risk groups. This analysis used only TCGA expression data and ranked 19,423 unique gene symbols by the signed Welch t-statistic to compare high-risk and low-risk patients in the TCGA cohort. Positive normalized enrichment scores indicate enrichment toward the high-risk group, whereas negative scores indicate enrichment toward the low-risk group. The analysis tested 11,327 gene sets, identifying 156 terms at FDR < 0.05 and 1,845 terms at FDR < 0.25.

**Fig. 5:**
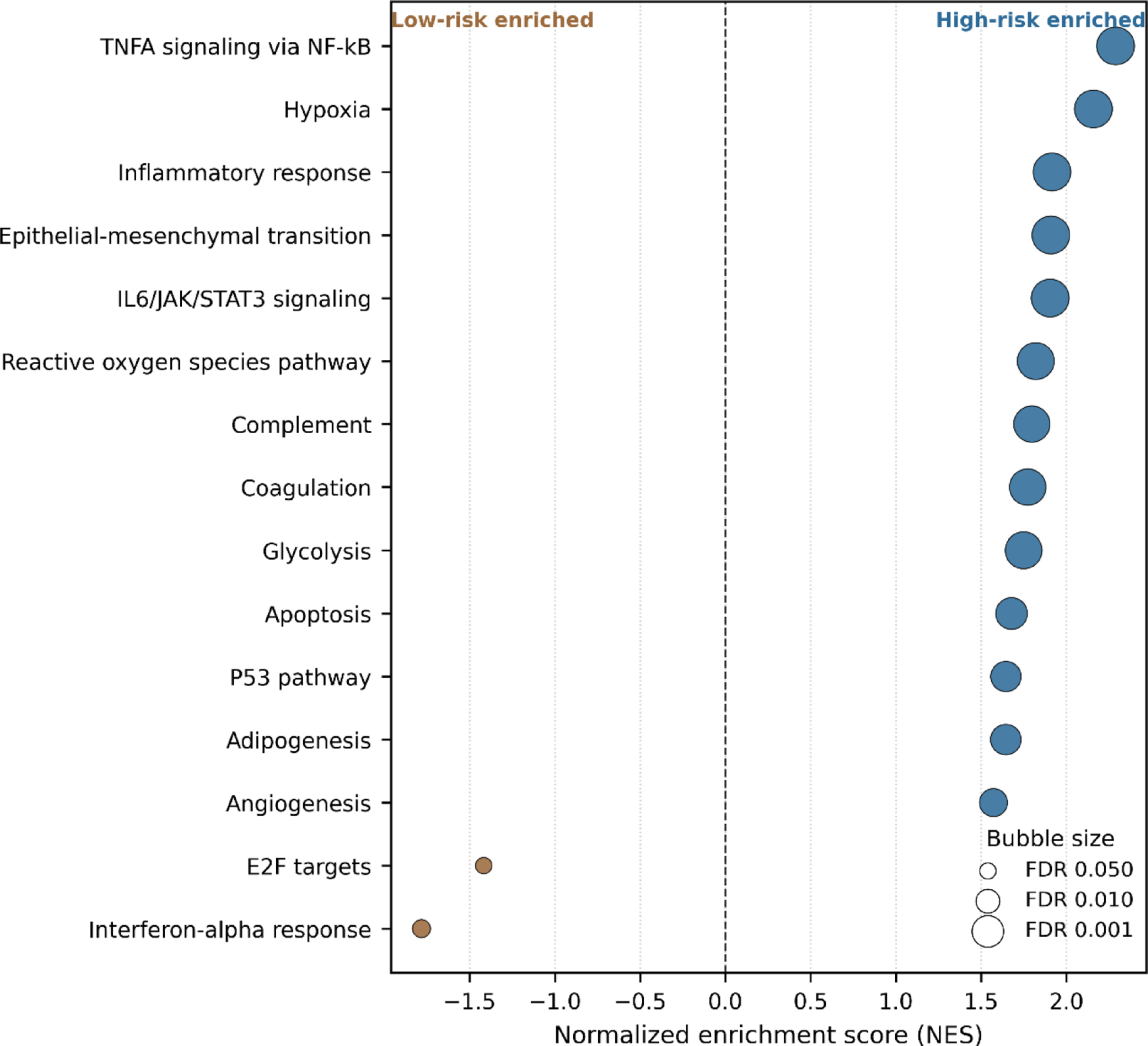
Hallmark GSEA of TCGA risk groups. Post-lock full-transcriptome TCGA preranked GSEA of median-OS risk groups. The analysis ranked 19,423 unique gene symbols by the signed Welch t-statistic when high-risk versus low-risk TCGA groups were compared, and 11,327 gene sets were tested. Positive normalized enrichment scores indicate high-risk enrichment, whereas negative scores indicate low-risk enrichment. The figure summarizes selected Hallmark terms at FDR < 0.05. High-risk enrichment included TNFA/NF-kB signaling, hypoxia, the inflammatory response, epithelial-mesenchymal transition, IL6/JAK/STAT3 signaling, the reactive oxygen species pathway, complement, coagulation, glycolysis, angiogenesis, and extracellular-matrix remodeling. Low-risk enrichment included the interferon-alpha response and E2F targets. GSEA was used for post-lock pathway contextualization, not for feature selection, threshold selection, external validation design, recalibration, refitting, classifier adjustment, or model validation.

Hallmark GSEA revealed a coherent high-risk enrichment pattern involving inflammatory, hypoxic, metabolic, and tissue-remodeling programs. The strongest positive Hallmark enrichments included TNFA signaling via NF-kB (NES = 2.289, FDR < 0.001) and hypoxia (NES = 2.159, FDR < 0.001), followed by the inflammatory response, epithelial-mesenchymal transition, IL6/JAK/STAT3 signaling, the reactive oxygen species pathway, complement, coagulation, glycolysis, and angiogenesis. Low-risk enrichment included the interferon-alpha response and E2F targets. Figure 5 summarizes selected Hallmark terms at FDR < 0.05.

The canonical pathway results provide additional support for related high-risk biological pathways, including the Reactome interleukin-10 signaling pathway, lysosome-related programs, and HIF1-associated pathway activity. These findings were interpreted as post-lock pathway-level contextualization of the TCGA risk groups. They were not used for feature selection, model tuning, threshold selection, external validation design, or any analytical decision affecting the locked classifier.

### Locked-signature ORA, Enrichr cross-checks, and PPI threshold sensitivity

Locked-signature ORA and Enrichr-style analyses were used as supplementary pathway cross-checks for the final signature and graph-associated locked genes. These analyses were placed below full-transcriptome GSEA in the evidence hierarchy because they operate on smaller selected gene sets and are therefore more sensitive to list composition. The most consistent locked-signature signals involved extracellular matrix organization, collagen formation, and related remodeling programs. External enrichment cross-checks of the locked all-unique genes and the locked WPPI-associated genes further supported ECM/collagen remodeling, lipid/lipoprotein metabolism, the cholesterol/PPAR-related context, and complement/coagulation-related pathway themes. Locked-signature ORA and supplementary GSEA visualizations are provided in Supplementary Figures S7 and S8.

A post-lock PPI confidence-threshold sensitivity audit assessed graph coverage at STRING score thresholds of 400, 700, and 900. At a STRING score ≥ 700, the audit retained 473,860 edges, preserved graph eligibility for all 58 locked WPPI-associated genes, and retained graph eligibility for 78 of 90 unique locked genes. By comparison, a STRING score ≥ 400 was more permissive and retained 1,858,944 edges, whereas a STRING score ≥ 900 was substantially sparser, retaining 201,712 edges and reducing locked WPPI-associated gene coverage to 40 of 58 genes. These results support a STRING score ≥ 700 as a high-confidence, coverage-preserving graph-construction cutoff. The audit was not used to retrain models, reselect features, change thresholds, or optimize CGGA performance. The complete PPI threshold sensitivity audit is provided in Supplementary Table S3.

## Discussion

This study developed GIBD as a locked, transcriptomics-only, graph-informed risk-prioritization framework for primary glioblastoma. The main implication is not that the model is clinically deployable, but that a TCGA-locked transcriptomic signal retained moderate external discrimination in CGGA while preserving a transparent and leakage-controlled development structure. This separation is particularly important in high-dimensional omics prediction, where feature selection, model selection, or threshold selection outside the validation structure can inflate performance estimates^8,29^. The final GIBD-XGBoost K100 model achieved CGGA AUC 0.609, sensitivity 73.9%, specificity 50.6%, balanced accuracy 62.3%, and C-index 0.537. These values indicate a measurable external signal rather than high predictive accuracy. Within the broader computational biology landscape, this graph-informed feature-engineering strategy is positioned as a transparent network-aware framework rather than as a graph neural network or heterogeneous-information-network learner^30–32^.

The practical significance of this result is therefore specific and interpretable. GIBD may be useful as a research-level transcriptomic prioritization score for identifying patients whose expression profiles resemble shorter-survival TCGA risk patterns. It should not be used as a standalone clinical decision rule, treatment-allocation tool, or calibrated individual risk calculator. The calibration and decision-curve analyses reinforce this interpretation: the score retained discrimination, but these analyses did not support using the locked score as a calibrated clinical probability estimate or as a stand-alone decision rule at the locked threshold^33–35^.

The ablation results further suggest that the final model represented a trade-off rather than universal dominance. Raw-RF produced higher sensitivity but low specificity, whereas GIBD-RF improved specificity but reduced sensitivity. The locked GIBD-XGBoost K100 model was retained because it provided a compact graph-informed representation with the most favorable balanced external operating profile among the main K100 comparators at the pre-specified threshold. These patterns are descriptive and should not be interpreted as statistically significant pairwise superiority.

### Methodological significance of TCGA-only locking and post-lock CGGA validation

A major strength of the workflow is its strict separation between development and external evaluation. TCGA was used for endpoint definition, feature construction, fold-contained feature selection, model selection, hyperparameter fixation, threshold selection, and final locking. The CGGA was introduced only after the feature set, model, scaler, and threshold were frozen. This structure is particularly important for transcriptomic glioblastoma modeling, where the number of candidate molecular variables is large relative to the number of available well-curated primary GBM samples. In such settings, even subtle reuse of external labels can inflate apparent generalizability.

The final CGGA evaluation therefore functions as a post-lock transportability assessment rather than as a second development phase. This distinction is central to interpreting the external results. The CGGA AUC of 0.609 and balanced accuracy of 62.3% are not presented as proof of clinical readiness. Instead, they show that the locked transcriptomic signal retained measurable discrimination in an independent cohort. The external operating profile was sensitivity-prioritized by design, reflecting the TCGA-derived threshold rule, and the moderate specificity should be interpreted as the expected trade-off of that operating point. This risk-prioritization profile would require further calibration, prospective testing, and clinical decision-analytic validation before any clinical deployment could be considered.

The detailed ablation results are therefore interpreted as descriptive evidence of external operating trade-offs rather than as proof of statistically significant superiority.

### WPPI-self features and graph-informed transcriptomic representation

WPPI-self feature construction was used to incorporate the network context while preserving the target-gene expression signal. Unlike simple unweighted neighborhood aggregation, the WPPI representation uses STRING v12 combined scores as confidence weights, excludes unmeasured or unmapped neighbors rather than zero-filling them, requires at least two valid measured non-self neighbors, and generates self-preserving features at three pre-specified alpha values. This design aligns more closely with the intended biological premise: gene expression should not be treated as isolated from the local network context, but neither should network aggregation erase the intrinsic expression of the target gene.

The role of STRING must be interpreted carefully. In GIBD, STRING is used as an external topology-and-confidence prior. It does not represent patient-specific proteomic abundance, patient-specific protein interaction activity, or direct evidence of functional PPI rewiring in individual tumors. Therefore, WPPI-derived features should be understood as graph-informed transcriptomic transformations, not as measured proteomic variables. This distinction protects the interpretation from overclaiming and is especially important because the model remains transcriptomics-only.

The final feature composition suggests that graph-informed representations were materially represented in the locked model: 65 of the 100 locked features were WPPI-derived. Post-lock SHAP analysis also revealed that WPPI-derived variables were among the top contributors. These observations support the methodological value of retaining graph-informed features in the learned model structure. However, they do not establish that specific PPI edges are causal, patient-specific, or biologically validated. They indicate that the model used graph-informed transcriptomic representations as part of its predictive structure.

The STRING combined-score cutoff of ≥ 700 was used as a high-confidence graph-construction threshold rather than as a post hoc performance-optimized threshold. The post-lock threshold audit showed that ≥ 400 was more permissive and substantially denser, whereas ≥ 900 was more stringent but reduced locked WPPI-associated gene coverage. The ≥ 700 cutoff therefore provided a balance between confidence and coverage, preserving graph eligibility for all locked WPPI-associated genes while reducing the edge set relative to the more permissive threshold.

This approach also fits within a broader trend in computational biology toward network-aware and graph-informed representation learning. Heterogeneous information network models and geometric deep learning frameworks have increasingly been used to integrate molecular entities, biological priors, and network structures in biomedical prediction tasks^30–32^. Those studies provide useful context for why neighborhood-level or non-Euclidean structure can be informative in biological machine learning. Nevertheless, GIBD is not a graph neural network or heterogeneous information network model, and the present study does not benchmark against those approaches. Instead, GIBD represents a computationally transparent graph-informed feature-engineering strategy that may be extended in future work when larger, harmonized, multimodal glioblastoma cohorts are available.

### External validation, calibration, and clinical-context interpretation

The external validation results support an externally retained transcriptomic risk signal, whereas calibration and decision curve analyses define the limits of immediate clinical interpretation. The final model preserved discrimination in CGGA; however, calibration analysis revealed that the predicted probabilities should not be treated as directly calibrated clinical risk estimates^33,34^. In both the TCGA OOF and CGGA external evaluations, the Brier score was worse than the corresponding prevalence-null Brier score, indicating that probability calibration remains imperfect. This finding limits the interpretation of absolute risk probabilities and reinforces the view that GIBD should be presented as a risk-prioritization score rather than as a calibrated clinical probability model.

Decision curve analysis provides a similar caution^35^. Internally, the model showed favorable net benefits relative to the treat-all and treat-none strategies at the locked threshold. In the CGGA, the model remained favorable relative to treat-all at the locked threshold but not relative to treat-none, with more favorable external decision curve behavior at lower threshold probabilities. This does not invalidate the transcriptomic signal, but it prevents any claim that clinical utility is proven at the locked operating threshold. The decision curve results are therefore best interpreted in a supportive context rather than as evidence for immediate clinical implementation.

Clinical-covariate benchmarking also placed the transcriptomic model in context. Age-only and age-plus-sex baselines showed comparable or slightly higher AUCs in CGGA, but their locked-threshold operating profiles had very low high-risk sensitivity. The exploratory GIBD-plus-age-plus-sex model improved the AUC and balanced accuracy, suggesting that transcriptomic and clinical covariates may carry complementary information. However, this combined model was not the final classifier and did not alter the locked transcriptomic model. Future work should evaluate whether integrated clinical-transcriptomic models improve calibrated risk prediction in larger cohorts with harmonized covariates, including MGMT promoter methylation, extent of resection, treatment details, steroid exposure, performance status, and imaging-derived tumor burden^36^.

### Model explanation and pathway contextualization

Post-lock SHAP and LIME analyses were used to interpret the frozen model, not to select features or validate biomarkers^18,19^. The global SHAP results identified *TSPAN13* as the strongest feature-level contributor, followed by a mixture of raw-expression and WPPI-derived variables, including *FCRL2* (WPPI-self25), *IFIT3*, *HOXD11* (WPPI-self25), *LYPD3* (WPPI-self75), *SREBF1*, *CTSA* (WPPI-self25), *HPS1* (WPPI-self50), *HOXC10* (WPPI-self75), and *GGT6* (WPPI-self50). Independent GBM/glioma literature provides concordant biological context for selected contributors, including *TSPAN13*^37^, HOX-family genes^38,39^, *CTSA*/cathepsin biology^40^, lipid-metabolic *SREBF1* context^41^, and interferon-related signals^42^. The presence of WPPI-derived features among the top contributors supports the relevance of the graph-informed representation to the learned model structure. However, SHAP values represent model-level attribution, not causal biology, biomarker validation, or proof that the corresponding genes drive patient outcome.

The supplementary SHAP interaction and dependence analyses should be interpreted in the same restrained manner. They show model-level nonlinear dependencies, not biochemical interactions or protein–protein interaction (PPI) validation^18^. This distinction is essential because tree-based interaction values can reveal how the model combines variables but cannot establish a molecular mechanism. Similarly, LIME examples in CGGA provide local explanations for representative true-positive, false-positive, true-negative, and false-negative cases^19^. Near-threshold false-positive and false-negative cases are useful for illustrating local decision-boundary uncertainty, but LIME intervals should not be interpreted as biological expression cutoffs.

The pathway analyses provide a separate biological context layer. The full-transcriptome TCGA preranked GSEA was treated as the primary pathway-contextualization analysis because it uses the fully measured transcriptome rather than only the selected model features^20,21^. High-risk TCGA samples were enriched for inflammatory, hypoxic, metabolic, and tissue-remodeling programs, including TNFA signaling via NF-kB, hypoxia, the inflammatory response, epithelial-mesenchymal transition, IL6/JAK/STAT3 signaling, the reactive oxygen species pathway, complement, coagulation, glycolysis, and angiogenesis. These programs are biologically plausible in aggressive glioblastoma, but they should be described as pathway-level contextualization of the TCGA risk groups, not as validation of the classifier^20,21^.

Locked-signature ORA and Enrichr-style cross-checks were retained as supplementary analyses below full-transcriptome GSEA in the evidence hierarchy, using curated pathway resources and enrichment tools, including Reactome and Enrichr^22,23^. The strongest coherent signals involved extracellular matrix organization, collagen formation, lipid/lipoprotein metabolism, cholesterol/PPAR-related context, and complement/coagulation-related themes. These signals are broadly concordant with a risk phenotype involving remodeling, inflammation, metabolic stress, and microenvironmental interactions. However, because ORA and Enrichr operate on selected gene lists, they are more sensitive to list composition and database content. They are therefore best framed as supplementary pathway contextualization rather than validation.

### Broader computational oncology and network biomarker modeling

GIBD fits within a broader computational oncology literature that seeks to convert high-dimensional molecular data into interpretable prognostic signatures. Selected cross-cancer studies illustrate this wider methodological landscape. RAD21-PON1 multi-omics analysis in hepatocellular carcinoma^10^, DNA methylation-driven DEG risk scoring in colorectal cancer^11^, and ceRNA network analysis in stomach adenocarcinoma^12^ highlight the increasing use of public molecular cohorts, regulatory networks, and multilayer molecular features to identify prognostic structures. These studies do not provide direct evidence for glioblastoma and do not validate GIBD. Rather, they support the broader premise that prognostic modeling is moving beyond isolated single-gene markers toward integrated molecular systems.

Within this landscape, GIBD occupies a specific niche. It is not a full multi-omics model, a graph neural network, or a heterogeneous information network learner. Instead, it uses a transparent graph-informed feature-engineering layer built on RNA-seq and STRING topology, followed by a locked nonlinear classifier. This design trades the representational flexibility of deeper graph models for interpretability, reproducibility, and clearer separation between development and validation. This trade-off is appropriate for the current sample size, where methodological transparency and leakage control are priorities.

Future versions of GIBD could explore more expressive network-learning strategies, including heterogeneous molecular networks and graph neural architectures^30–32^, but such extensions would require larger training cohorts, independent validation, careful calibration, and stronger safeguards against overfitting. The present study should therefore be viewed as a reproducible graph-informed transcriptomic framework that can support future, more expressive network-based glioblastoma modeling as larger harmonized cohorts become available.

### Relationship to multimodal glioblastoma heterogeneity literature

Glioblastoma heterogeneity is not limited to transcriptomics. It is spatial, metabolic, radiographic, histologic, immunologic, and treatment-response dependent. Selected imaging and spectroscopy studies clarify this point. MRI texture analyses have shown that local and regional imaging heterogeneity can be associated with survival stratification^24^. MRI-derived shape and intensity frameworks have further emphasized that tumor morphology and intensity patterns can identify groups with distinct survival profiles^25^. MR-spectroscopy studies highlight metabolic heterogeneity and the potential for noninvasive molecular characterization^26^. More recent multimodal imaging work has examined spatial response heterogeneity in glioblastoma, including regional differentiation of true progression and pseudoprogression^27,28^.

These studies provide important context for interpreting GIBD. Our model captures a transcriptomic risk-prioritization layer, not the full biology of glioblastoma heterogeneity. It does not use imaging-texture features^24^, tumor shape-and-intensity descriptors^25^, MR-spectroscopy or metabolic-imaging information^26,27^, or spatial response-assessment features^28^. Therefore, it should not be positioned as a replacement for neuroimaging, histopathology, multidisciplinary clinical evaluation, or treatment‒response assessment. Instead, it may be viewed as a molecular component that could eventually complement multimodal risk models.

This distinction also explains why direct performance comparisons between GIBD and imaging or spectroscopy studies would be inappropriate. The modalities, endpoints, patient populations, spatial units of analysis, and validation designs differ substantially. Some imaging studies classify voxels or supervoxels; others stratify patients using imaging-derived clusters or response labels. GIBD performs patient-level transcriptomic median-OS risk classification. These are related but not interchangeable prediction problems, as the cited imaging, spectroscopy, metabolic, and spatial-response studies use modality-specific inputs, analysis units, endpoints, and validation designs^24–28^. The appropriate conclusion is that transcriptomic, imaging, and metabolic heterogeneity each capture different aspects of glioblastoma biology, and future models should evaluate whether their integration improves robust, calibrated, and clinically meaningful prognostication.

### Limitations

Several limitations must be acknowledged. First, the study used retrospectively assembled public transcriptomic cohorts. This design enabled reproducible TCGA-only development and independent post-lock CGGA external validation, but it does not replace prospective clinical evaluation. Second, the TCGA development cohort remains small relative to the transcriptomic feature space, a common but important limitation in glioblastoma omics modeling^29^. The use of fold-contained feature selection, regularized XGBoost, and a frozen external validation design mitigates this risk but cannot eliminate it.

Third, the external validation results demonstrated an externally retained prognostic signal under independent CGGA evaluation, but the performance remains insufficient for clinical deployment. The CGGA AUC of 0.609 and C-index of 0.537 should be interpreted as moderate discrimination and limited survival-ranking concordance under post-lock external validation, not as evidence of a clinically validated predictor. Specificity was moderate at the locked sensitivity-prioritized threshold, and probability calibration did not support direct clinical risk-probability interpretation^33,34^; therefore, prospective recalibration and multimodal validation are needed before translational use. Fourth, decision curve analysis did not establish external clinical utility at the locked threshold relative to treat-none^35^, reinforcing that the current model should be treated as a risk-prioritization signal rather than a clinical decision rule.

Fifth, the model is transcriptomics-only. It does not include important clinical and molecular variables such as *MGMT* promoter methylation, extent of resection, corticosteroid exposure, treatment adherence, performance status, or radiographic tumor burden, nor does it include multimodal heterogeneity measures such as imaging texture^24^, tumor shape-and-intensity descriptors^25^, MR-spectroscopy or metabolic-imaging information^26,27^, or spatial response-assessment features^28^. Sixth, the WPPI layer uses STRING topology as an external prior, not patient-specific proteomic measurements. This prevents interpretation of WPPI-derived features as direct evidence of patient-specific protein interaction activity^15^.

Seventh, interpretability and pathway analyses are not causal validation. SHAP and LIME describe model behavior^18,19^; GSEA, ORA, and Enrichr contextualize pathway patterns via gene set and pathway resources^20–23^; and the PPI threshold audit evaluates graph coverage via STRING topology^15^. None of these analyses proves that specific genes, pathways, or PPI edges causally drive survival. Finally, although the network-learning and multimodal heterogeneity literature motivates future development, the present study did not benchmark against graph-learning or heterogeneous-information-network models^30–32^, nor did it benchmark against multimodal imaging, metabolic, or spatial response-assessment models^24–28^.

### Future work

Future work should proceed in several directions. First, GIBD should be evaluated prospectively in multicenter cohorts with standardized RNA-seq processing and harmonized clinical annotation^36^. Second, probability calibration should be improved before any clinical decision-support use is considered. Third, transcriptomic risk scores should be evaluated alongside established clinical and molecular covariates, including age, performance status, MGMT promoter methylation, extent of resection, treatment variables, and radiographic tumor burden.

Fourth, multimodal integration should be prioritized. Imaging-texture features^24^, shape-and-intensity descriptors^25^, MR-spectroscopy and metabolic-imaging information^26,27^, and spatial response-assessment approaches^28^ may capture complementary aspects of glioblastoma heterogeneity that are not available from bulk transcriptomics alone. Fifth, graph-learning extensions may be considered once larger datasets become available. These methods could include heterogeneous molecular networks, graph neural networks, attention-based neighborhood weighting, or multi-omics graph representations^30–32^. However, such models should be developed only under rigorous external validation, calibration assessment, and transparent reporting to avoid trading interpretability for apparent performance^36^.

## Conclusion

GIBD provides a locked, reproducible, graph-informed transcriptomic prognostic risk-prioritization approach for primary glioblastoma. By combining TCGA-only development, self-preserving WPPI feature construction, frozen XGBoost modeling, post-lock CGGA validation, and post-lock interpretability and pathway contextualization, the workflow addresses several key concerns in high-dimensional biomarker modeling. The final model preserved a measurable external risk signal and showed a sensitivity-prioritized operating profile in CGGA. Pathway analyses supported the biological plausibility of inflammatory, hypoxic, metabolic, remodeling, and coagulation-related programs. Nevertheless, the model should be interpreted as a computational risk-prioritization signal, not as a clinically validated decision tool or causal biological model. Prospective validation, recalibration, and multimodal assessment will be needed before any translational use can be considered.

## Methods

### Study design and external validation

We designed GIBD as a locked, transcriptomics-only prognostic risk-prioritization framework for primary glioblastoma. The workflow was structured to separate model development from external validation. TCGA was used for cohort curation, survival-endpoint definition, weighted protein–protein interaction (WPPI) feature construction, feature selection, candidate model selection, and threshold locking. CGGA was used as a post-lock external validation cohort.

CGGA labels were not inspected or used during feature construction, feature selection, model selection, hyperparameter selection, threshold selection, pathway ranking, scaling-parameter fitting, or final classifier locking. External CGGA labels were used only after locking to compute validation metrics and to label representative post hoc explanation cases. This design was intended to reduce information-leakage risk and preserve the CGGA as an independent post-lock external validation cohort.

### Cohort acquisition, eligibility criteria, and survival endpoint definition

RNA-seq expression and clinical survival data were obtained from the TCGA and CGGA^16,17^. The TCGA cohort served as the discovery cohort and model-locking cohort, whereas the CGGA cohort served as the post-lock external validation cohort. We retained primary glioblastoma cases with available overall-survival information and protein-coding transcriptomic profiles. When molecular annotation was available, cases inconsistent with primary IDH-wildtype glioblastoma were excluded from the analysis to align with contemporary CNS tumor classification^43^.

The final TCGA cohort included 147 patients. The external CGGA cohort contained 133 eligible patients before censored binary-evaluation filtering. The primary supervised endpoint was binary prognostic risk defined via the empirical TCGA median overall survival. Overall survival was measured in days. The median OS of the TCGA cohort was 357 days. TCGA patients with OS ≤ 357 days were labeled high risk, whereas TCGA patients with OS > 357 days were labeled low risk. This produced 74 high-risk and 73 low-risk TCGA patients.

The same 357-day cutoff was applied to the CGGA after model locking. CGGA patients with an observed event before or at 357 days were labeled high risk, and patients surviving beyond 357 days were labeled low risk. Patients censored before 357 days were considered binary-ambiguous and were excluded from binary AUC and locked-threshold classification metrics rather than being forced into artificial low-risk or high-risk categories. The final binary-evaluable CGGA set included 131 patients, including 46 high-risk patients and 85 low-risk patients. Survival time and censoring information were retained where applicable for secondary concordance-index analysis.

### RNA-seq preprocessing and expression matrix construction

The RNA-seq expression values were processed into log-CPM-normalized expression matrices. Gene identifiers were harmonized by removing version suffixes where present, duplicate gene columns were removed, and numeric expression matrices were used for downstream analysis. WPPI features were generated de novo from log-CPM-normalized measured gene expression values and STRING v12 topology before model fitting. WPPI construction uses external STRING topology only and does not use patient-specific proteomic measurements, CGGA labels, or CGGA outcome information. Candidate raw expression and WPPI-derived features were then subjected to TCGA-only feature selection, with feature ranking recomputed within each TCGA training fold during out-of-fold evaluation.

For model fitting, continuous features were standardized using scaling parameters estimated from TCGA only. During out-of-fold evaluation, the scaler was fitted within each TCGA training fold and applied to the corresponding held-out TCGA fold. After final model locking, the scaler was refitted on the full TCGA development cohort and then applied unchanged to the aligned CGGA matrix. No CGGA labels or CGGA-derived outcome information were used to fit scaling parameters, select features, or modify the final feature order.

For post-lock external validation, the CGGA expression matrix was aligned to the locked TCGA feature space. The same frozen feature list and feature order were used for CGGA scoring. No external outcome information was used to create, select, substitute, or modify features.

### Weighted PPI self-preserving feature construction

We constructed a weighted PPI self-preserving representation to integrate target-gene expression with confidence-weighted network-neighbor information. STRING v12 protein–protein interaction topology was used as an external graph prior^15^. STRING edges were retained using a high-confidence combined score cutoff of ≥ 700. The STRING graph was used to define topology and confidence weights only; it was not interpreted as measured patient-specific protein abundance or patient-specific proteomic interaction activity^13,15^.

Let ${\mathcal{G}}_{x}$ denote the set of genes measured in the expression matrix, let ${x}_{g}$ denote the expression vector of target gene $g$ across patients, and let ${x}_{j}$ denote the expression vector of neighboring gene $j$. Let $P\left(g\right)$ denote the set of STRING protein identifiers mapped to gene $g$, and let ${s}_{pq}$ denote the STRING combined score for protein pair $\left(p,q\right)$. For each target gene $g$, only valid non-self measured neighbor genes were aggregated. The valid measured neighbor set was defined as:

$${\mathcal{N}}_{\mathcal{g}}=\{j\in {\mathcal{G}}_{\mathcal{X}}: j\ne g, {a}_{gj}>0\}$$

The gene-level confidence weight ${a}_{gj}$ between the target gene $g$ and neighboring gene $j$ was computed by accumulating scaled STRING combined scores across retained protein–protein mappings connecting proteins assigned to $g$ and proteins assigned to $j$, where ${s}_{pq}$ denotes the STRING combined score for protein pair $\left(p,q\right)$:

$${a}_{gj}={\sum}_{\genfrac{}{}{0pt}{}{p\in P\left(g\right), q\in P\left(j\right)}{{s}_{pq}\ge 700}}\frac{{s}_{pq}}{1000}$$

The confidence-weighted neighbor signal for the target gene $g$ was then computed as the weighted average of the measured neighbor-gene expression vectors:

$$Nb{r}_{w}\left(g\right)=\frac{{\sum}_{j\in {\mathcal{N}}_{\mathcal{g}}}{a}_{gj}{x}_{j}}{{\sum}_{j\in {\mathcal{N}}_{\mathcal{g}}}{a}_{gj}}$$

Unmapped or unmeasured neighbor genes were excluded rather than zero-filled. WPPI-self features were generated only when at least two valid measured non-self neighbors were available:

$$\left|{\mathcal{N}}_{\mathcal{g}}\right|\ge 2$$

To preserve the intrinsic expression of the target gene while incorporating a weighted graph context, the self-preserving WPPI feature was defined as:

$$WPP{I}_{\alpha }\left(g\right)=\alpha {x}_{g}+\left(1-\alpha \right)Nb{r}_{w}\left(g\right),\hspace{1em}\alpha \in \{0.25, 0.50, 0.75\}$$

This yielded three candidate WPPI-self features per eligible gene. Pure weighted-neighbor-only features were not included in the final locked feature space. The final locked K = 100 model contained 65 WPPI-derived features and 35 raw-expression features. The algorithm details for WPPI-self feature construction, including STRING edge filtering, valid measured neighbor selection, gene-level confidence-weight accumulation, minimum-neighbor handling, and alpha-specific feature generation, are provided in Supplementary Table S1.

### Feature selection, model development, and TCGA-only model locking

Feature selection and model locking were performed using TCGA only. Random-forest Gini importance was used as a nonlinear feature-ranking mechanism, not as the final classifier^44^. Within repeated cross-validation, feature selection was recomputed inside each TCGA training fold to avoid selecting features via held-out fold labels.

The candidate models were evaluated via repeated stratified fivefold cross-validation with four repeats. This produced 20 validation folds. Each TCGA patient received out-of-fold predictions across the repeated validation process, and per-patient OOF probabilities were averaged when a patient appeared in multiple held-out folds.

The final model was a frozen GIBD-XGBoost classifier with K = 100 locked features. The final XGBoost configuration used max_depth = 3, learning_rate = 0.029, n_estimators = 85, gamma = 0.18, reg_alpha = 0.30, reg_lambda = 6.5, scale_pos_weight = 2.4, subsample = 0.80, colsample_bytree = 0.75, and min_child_weight = 1.8^45^. XGBoost was selected as the final classifier because it is well suited to tabular high-dimensional biomedical data, can model nonlinear feature effects and feature interactions, and provides regularization controls for tree complexity, shrinkage, subsampling, and split penalties. Random forest and LASSO-Cox models were retained as comparator model families to assess whether the final operating profile depended on the tree-boosting model family.

Hyperparameter handling was constrained and TCGA-only rather than implemented as a broad nested hyperparameter search. The final GIBD-XGBoost K100 model used one fixed XGBoost configuration, reported above and summarized in Supplementary Table S2, during TCGA out-of-fold evaluation and subsequent model locking; therefore, no multi-configuration hyperparameter search was performed within validation folds. Within TCGA cross-validation, feature selection, scaler fitting, model fitting, and out-of-fold prediction generation were recomputed fold-wise. TCGA out-of-fold estimates are therefore interpreted as internal development estimates for this fixed configuration, not as estimates from a fully nested hyperparameter-optimization procedure.

The final classification threshold was selected from TCGA out-of-fold probabilities via the pre-specified recall80_spec25 rule, which prioritized high-risk sensitivity while requiring minimum specificity support. The selected threshold was 0.53 and was fixed before CGGA evaluation. After locking, CGGA results were not used for feature reselection, hyperparameter selection or adjustment, threshold adjustment, recalibration, refitting, or any change to the locked classifier. The final configuration, random seeds, package versions, locked artifacts, and script run order are summarized in Supplementary Table S2.

### Ablation and comparator analyses

Ablation analyses were designed to isolate graph-informed feature constructions, model-family effects, and feature-budget complexity. All comparator models were developed under TCGA-only fold-contained procedures and then evaluated via CGGA only after their features, parameters, and operating thresholds were fixed using TCGA-only information.

The graph-impact comparison contrasted the locked WPPI-informed GIBD-XGBoost K = 100 model with a raw-expression XGBoost K = 100 baseline. Model-family comparisons included raw-expression random forest, GIBD-random forest, and raw-expression LASSO-Cox baselines^9,44–46^. A GIBD-XGBoost K = 120 complexity-control comparator was included to assess whether a larger graph-informed feature budget improved the external trade-off. The CGGA was introduced only after all the candidate models and thresholds had been fixed.

### External validation in the CGGA

The final frozen GIBD-XGBoost model was evaluated on the CGGA in a single post-lock external validation pass. Primary discrimination was assessed via the AUC. The AUPRC was also reported to account for class distribution. Threshold-based performance was evaluated at the locked threshold of 0.53 via sensitivity, specificity, balanced accuracy, and the Matthews correlation coefficient. The concordance index was reported as a secondary survival-ranking metric where OS and censoring information were available^46^.

The external analysis was interpreted as a transportability assessment under cohort and sequencing-domain shift, not as prospective clinical validation. CGGA performance results were used only for post-lock reporting and were not used to change the locked classifier.

### Calibration, decision curve analysis, and clinical-covariate benchmarking

Post-lock clinical-context analyses were performed to characterize probability calibration and decision-analytic behavior. Calibration was assessed via the Brier score, Brier improvement relative to prevalence, and expected calibration error^33,34^. Decision curve analysis was used to compare model net benefit against treat-all and treat-none strategies across threshold probabilities^35^. DCA was interpreted as range-based supportive evidence and not as proof of clinical utility at the locked classification threshold.

A supplementary clinical-covariate benchmark was added to contextualize the transcriptomics-only GIBD score. Age-only and age-plus-sex logistic baselines were evaluated as clinical comparators. For these clinical baselines, thresholds were derived from TCGA development data and did not affect the locked GIBD threshold. An exploratory GIBD-score-plus-age-plus-sex model was also evaluated to assess whether clinical covariates might provide complementary information. This combined model was treated as a supplementary contextual analysis only; it was not used as the final classifier and did not alter the frozen GIBD-XGBoost model.

### Post-lock explainability via SHAP and LIME

SHAP analysis was performed post hoc on the final frozen K = 100 GIBD-XGBoost model. SHAP was used to summarize global feature contributions and identify influential raw-expression and WPPI-derived features in the locked model^18^. SHAP analysis was not used for feature selection, model tuning, threshold modification, pathway analysis, or biological validation.

LIME was used as a local post hoc explainability method for representative external CGGA cases selected from the frozen model predictions after model locking. The selected cases included true-positive, false-positive, true-negative, and false-negative examples. LIME-discretized intervals were interpreted as local model-explanation intervals, not as biological expression cutoffs^19^.

SHAP interaction values, where reported, were treated as exploratory model-level nonlinear dependency patterns rather than biochemical or molecular interactions.

### Pathway enrichment and biological contextualization

Pathway analyses were performed after model locking to evaluate biological plausibility and contextualize the final signature. These analyses were not used for feature selection, model tuning, threshold selection, external validation design, recalibration, refitting, or any change to the locked classifier.

The full-transcriptome TCGA preranked GSEA was treated as the primary pathway-contextualization analysis^20,21^. Genes were ranked by signed Welch t-statistics to compare the TCGA median-OS between the high-risk and low-risk groups. Positive normalized enrichment scores indicate enrichment toward the high-risk group, whereas negative scores indicate enrichment toward the low-risk group. The full-transcriptome GSEA analysis was interpreted as a post hoc biological plausibility analysis, not as model validation.

Overrepresentation analysis was performed on the final locked signature, WPPI-associated locked genes, and SHAP-ranked gene subsets via local GMT pathway collections, including Reactome and MSigDB-derived gene sets^21,22^. Enrichr-style analyses were used as complementary pathway contexts for locked all-unique genes, WPPI-associated genes, and SHAP top-gene subsets^23^. Locked-signature ORA and Enrichr-style analyses were treated as supplementary cross-checks below full-transcriptome GSEA in the pathway evidence hierarchy.

### PPI confidence threshold sensitivity audit

A post-lock PPI confidence threshold sensitivity audit evaluated how the selected STRING confidence threshold affected graph coverage^15^. STRING thresholds of 400, 700, and 900 were compared via expression‒gene graph eligibility and coverage of locked WPPI-associated genes. The audit was used to assess whether the selected STRING score threshold of 700 preserved the final locked WPPI-associated signature while reducing lower-confidence network density. The audit was interpreted as graph coverage sensitivity analysis and did not retrain models, reselect features, change thresholds, use CGGA labels, or optimize external performance.

### Statistical analysis and performance metrics

We evaluated discrimination via the AUC and AUPRC. We summarized the locked-threshold classification performance via sensitivity, specificity, balanced accuracy, and the Matthews correlation coefficient. We assessed calibration via the Brier score and the expected calibration error^33,34^. We reported the concordance index only as a secondary survival-ranking metric^46^ because the primary endpoint was the median-OS binary risk label.

For internal model-development estimates, we used TCGA out-of-fold predictions generated during repeated stratified cross-validation. For external validation, we applied the frozen feature set, TCGA-fitted scaler, frozen XGBoost classifier, and locked threshold to the CGGA in a single post-lock evaluation step. CGGA results were not used for recalibration, refitting, threshold adjustment, or any change to the locked classifier. Post hoc interpretive analyses were reported separately from predictive validation and were treated as non-causal biological contextualization.

### Software implementation and reproducibility

We implemented the analysis as a scripted Python workflow with separate stages for preprocessing, WPPI feature construction, TCGA-only model development, post-lock external validation, ablation testing, calibration and decision-curve analysis, clinical-covariate benchmarking, SHAP/LIME explainability, pathway analysis, and PPI threshold sensitivity auditing. The scripts were organized in a fixed run order, and stochastic procedures used fixed random seeds where applicable. The GSEA and ORA workflows used local GMT resources and GSEApy where applicable^47^. The final reproducibility configuration, including model hyperparameters, stochastic seeds, fixed script run order, locked feature artifacts, and post-lock analysis outputs, is summarized in Supplementary Table S2.

The final locked GIBD workflow was implemented using the Python programming language version 3.13.5 (https://www.python.org/) on a Microsoft Windows 11 operating system (https://www.microsoft.com/windows). Core software components used in the final numbered workflow included NumPy version 2.3.4 (https://numpy.org/), pandas version 2.3.3 (https://pandas.pydata.org/), SciPy version 1.16.2 (https://scipy.org/), scikit-learn version 1.8.0 (https://scikit-learn.org/), XGBoost version 3.0.5 (https://xgboost.readthedocs.io/), scikit-survival version 0.27.0 (https://scikit-survival.readthedocs.io/), SHAP version 0.49.1 (https://shap.readthedocs.io/), LIME version 0.2.0.1 (https://github.com/marcotcr/lime), GSEApy version 1.1.13 (https://gseapy.readthedocs.io/), Matplotlib version 3.10.7 (https://matplotlib.org/), and joblib version 1.5.2 (https://joblib.readthedocs.io/).

## Supplementary Information

Below is the link to the electronic supplementary material.

Supplementary Material 1

## Data Availability

The transcriptomic and clinical datasets analyzed in the present study are publicly available from the Genomic Data Commons Data Portal for TCGA data (https://portal.gdc.cancer.gov/) and the Chinese Glioma Genome Atlas database for CGGA data (http://www.cgga.org.cn/). The STRING protein–protein interaction data used for graph-informed feature construction are publicly available from the STRING databaseoma Genome Atlas database for CGGA data (http://www.cgga.org.cn/). The STRING protein–protein interaction data used for graph-informed feature construction are publicly available from the STRING database (https://string-db.org/). Gene-set resources used for pathway contextualization were obtained from publicly available pathway collections, including MSigDB/GMT and Reactome-associated gene-set resources, as described in the Methods.

## Abbreviations

AUC: Area under the receiver operating characteristic curve
AUPRC: Area under the precision‒recall curve
CGGA: Chinese glioma genome atlas
DCA: Decision curve analysis
ECM: Extracellular matrix
FDR: False discovery rate
GBM: Glioblastoma
GIBD: Graph-informed biomarker discovery
GSEA: Gene set enrichment analysis
IL6/JAK/STAT3: Interleukin-6/Janus kinase/signal transducer and activator of transcription 3
LIME: Local Interpretable model-agnostic explanations
MGMT: O6-methylguanine-DNA methyltransferase
NES: Normalized enrichment score
NF-kB: Nuclear factor kappa B
OOF: Out-of-fold
ORA: Overrepresentation analysis
OS: Overall survival
PPI: Protein–protein interaction
PPAR: Peroxisome proliferator-activated receptor
RNA-seq: RNA sequencing
SHAP: Shapley additive explanations
TCGA: The cancer genome atlas
TNFA: Tumor necrosis factor alpha
WPPI: Weighted protein–protein interaction
XGBoost: Extreme gradient boosting

## Code availability

The public-facing analysis scripts supporting the final locked GIBD workflow are available at: https://github.com/ossaamamahmoud/GIBD_Glioblastoma_Prognosis and archived in Zenodo with the versioned DOI: 10.5281/zenodo.20617146. The repository includes the numbered analysis scripts, run-order documentation, software requirements, README, and figure-generation documentation needed to reproduce the workflow when authorized input data are obtained from the original data sources.
