# Supplementary material for: Graph informed biomarker discovery framework using transcriptomic machine learning for glioblastoma prognosis
Source: Sci Rep. 2026 Jun 23;16:19530. doi: 10.1038/s41598-026-58062-4 (PMC13291226; doi:10.1038/s41598-026-58062-4)
Supplement: Supplementary file 1 — Supplementary Material 1 [file 41598_2026_58062_MOESM1_ESM.pdf]

Supplementary Information

Graph informed biomarker discovery framework using transcriptomic machine learning for glioblastoma prognosis

Osama Mahmoud[1*], Mahmoud Mounir[1] and Walaa Gad[1]

[1] Information Systems Department, Faculty of Computer and Information Sciences, Ain Shams University, Cairo, Egypt

*Correspondence: osama.mahmoud@cis.asu.edu.eg

Emails: Osama Mahmoud: osama.mahmoud@cis.asu.edu.eg

Mahmoud Mounir: mahmoud.mounir@cis.asu.edu.eg

Walaa Gad: walaagad@cis.asu.edu.eg

Supplementary Information contents

Supplementary Figure S1. Calibration analysis.

Supplementary Figure S2. Decision-curve analysis.

Supplementary Figure S3. Clinical-covariate benchmark.

Supplementary Figure S4. CGGA LIME local explanations.

Supplementary Figure S5. SHAP interaction analysis.

Supplementary Figure S6. SHAP targeted dependence analysis.

Supplementary Figure S7. Locked-signature ORA dotplots.

Supplementary Figure S8. Supplementary Hallmark GSEA / extended GSEA outputs.

Supplementary Table S1. WPPI-self feature construction pseudocode.

Supplementary Table S2. Reproducibility summary.

Supplementary Table S3. PPI threshold sensitivity audit.

Supplementary Figures

Supplementary Figure S1. Calibration analysis.

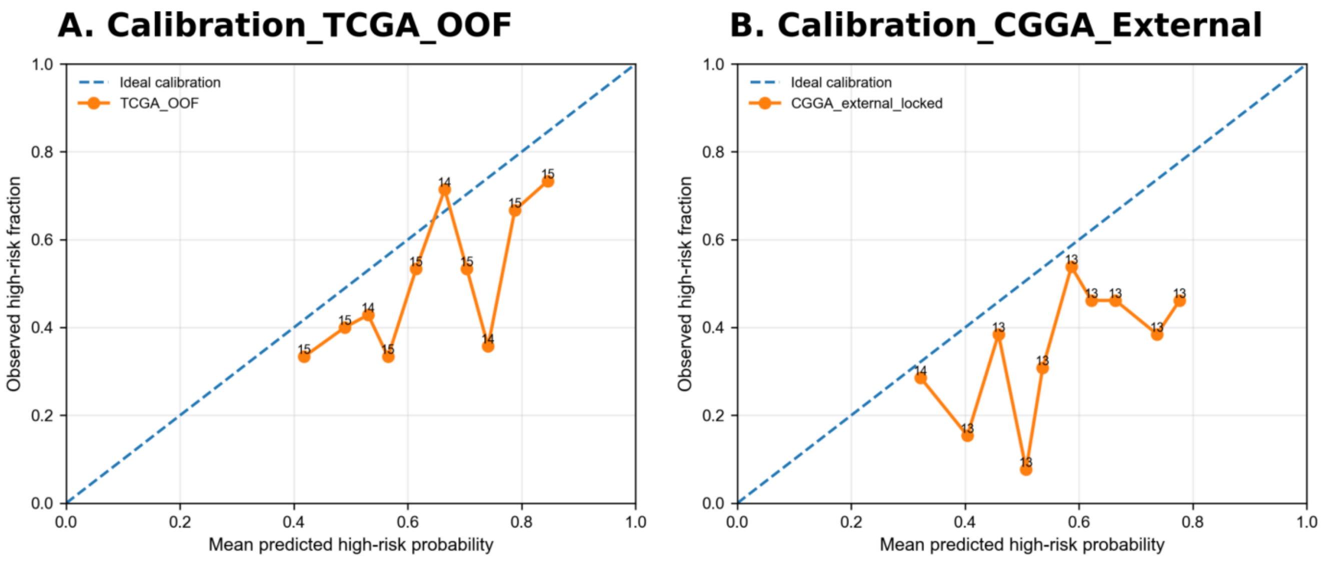

Legend. Calibration assessment of the locked GIBD-XGBoost K100 score in TCGA out-of-fold predictions and post-lock CGGA external validation. Calibration was interpreted as probability-calibration context and not as evidence of clinical deployment readiness.

Supplementary Figure S2. Decision-curve analysis.

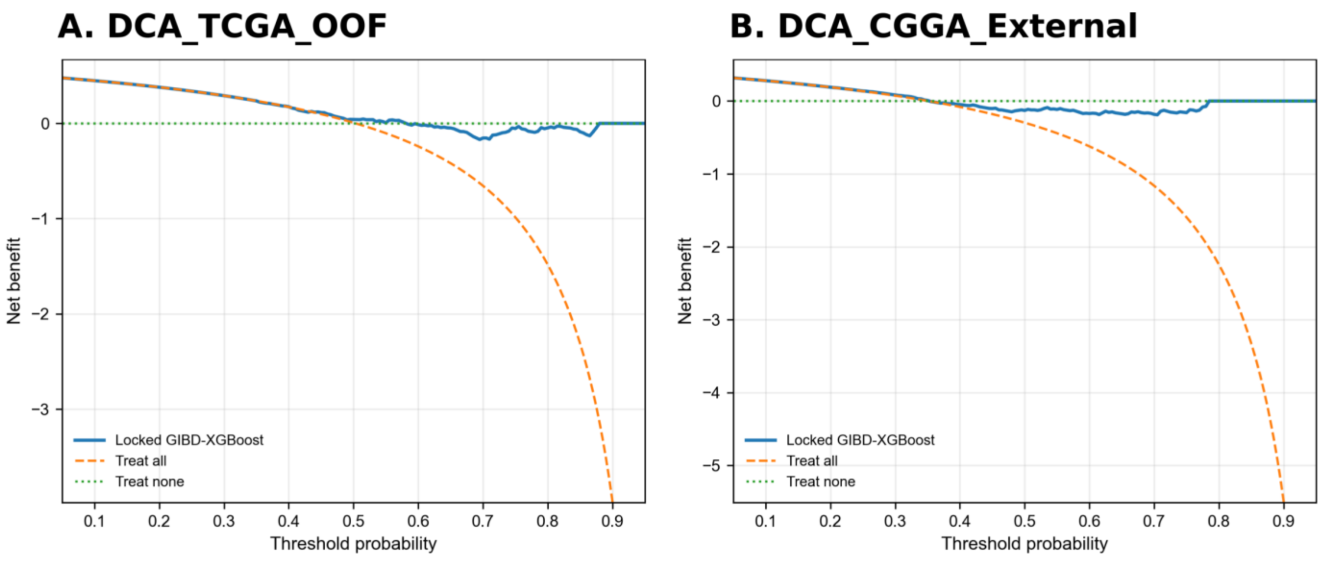

Legend. Decision-curve analysis comparing the locked GIBD-XGBoost K100 score with treat-all and treat-none strategies across threshold probabilities in TCGA out-of-fold and post-lock CGGA external evaluation. Decision-curve results were interpreted as supportive clinical-context analysis rather than proof of clinical utility.

Supplementary Figure S3. Clinical-covariate benchmark.

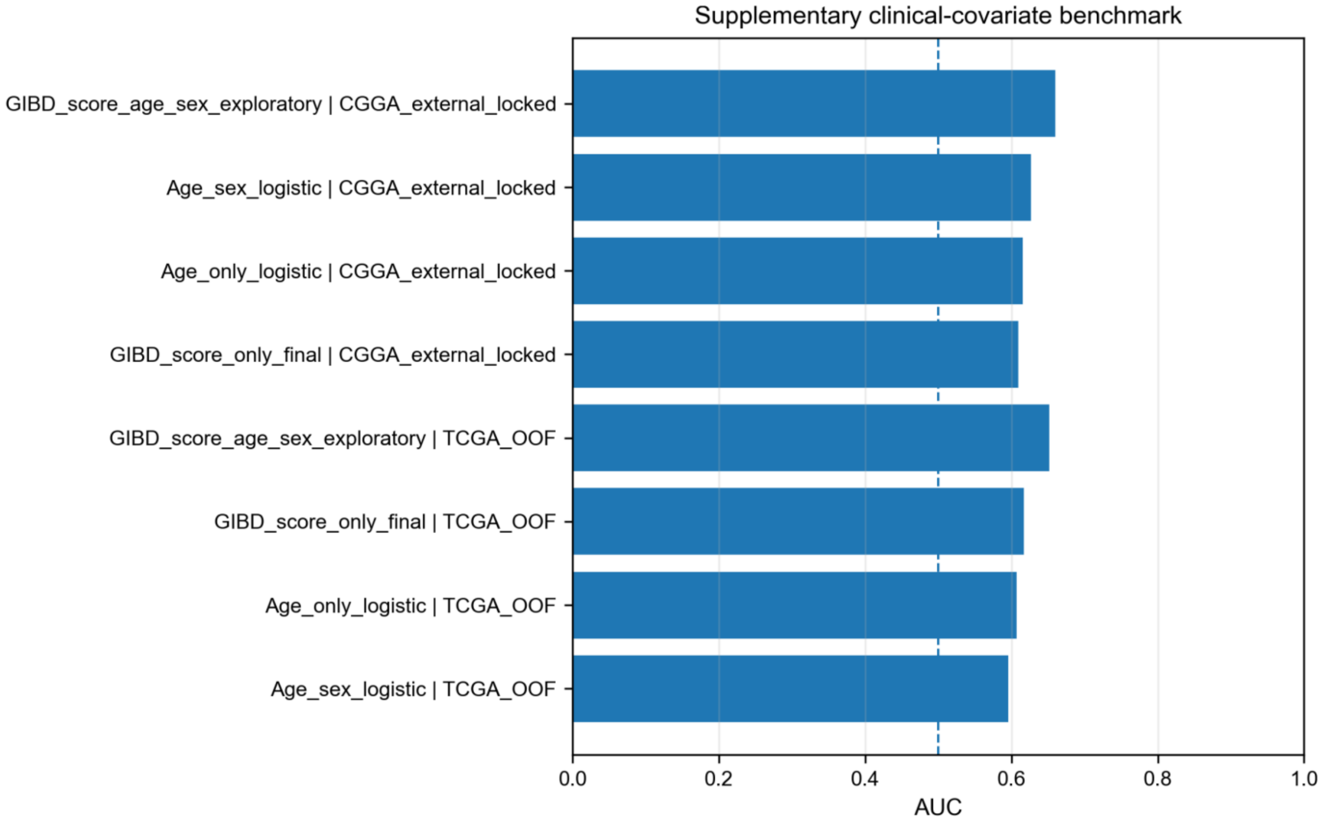

Legend. Clinical-covariate benchmark comparing age-only, age-plus-sex, locked GIBD-score-only, and exploratory GIBD-score-plus-age-plus-sex models. The combined clinical-transcriptomic model was treated as contextual only and did not alter the final locked transcriptomic classifier.

Supplementary Figure S4. CGGA LIME local explanations.

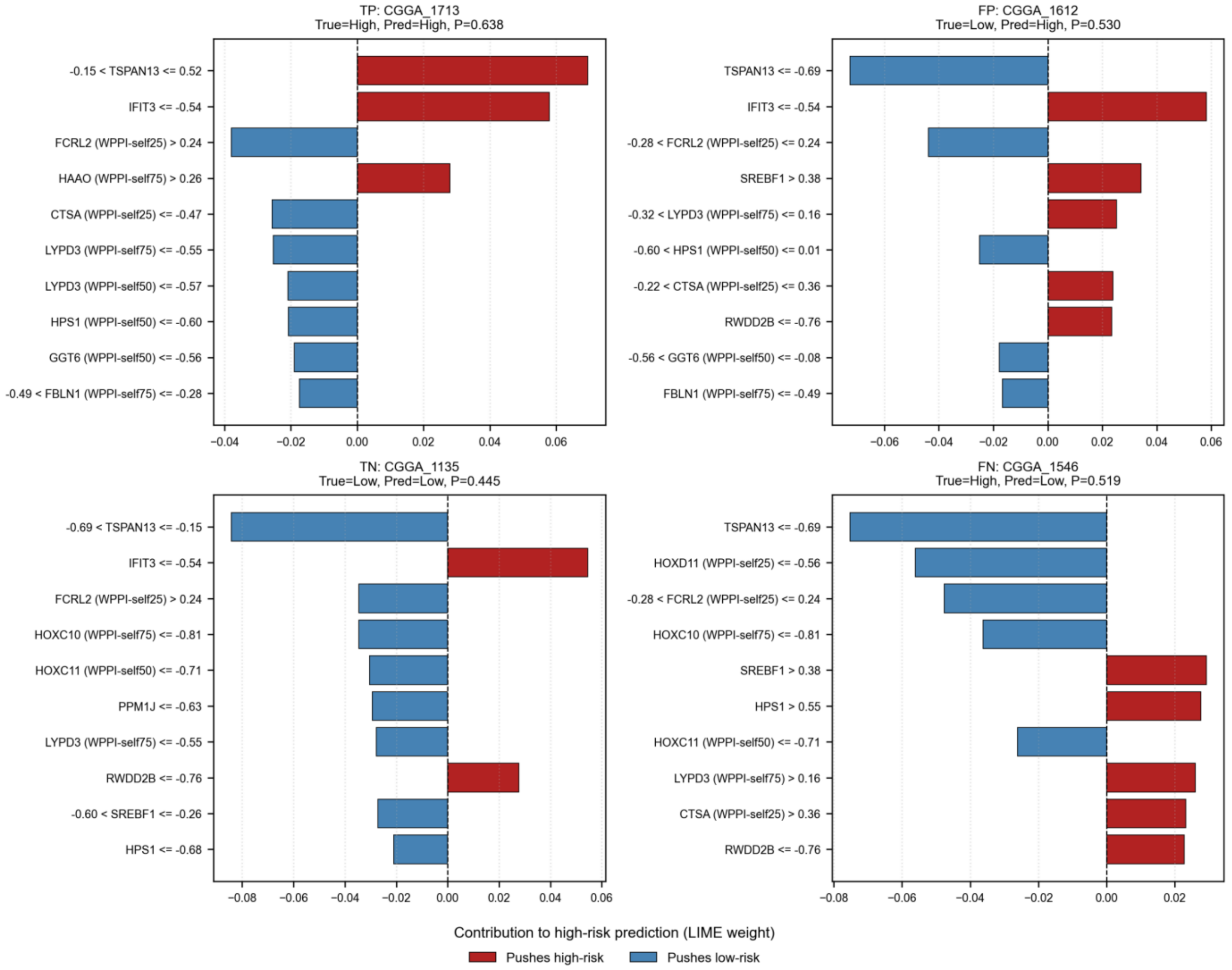

Legend. Post-lock LIME local explanations for representative CGGA external cases, including true-positive, false-positive, true-negative, and false-negative examples. LIME intervals represent local model-explanation intervals and should not be interpreted as biological expression cutoffs or biomarker validation.

Supplementary Figure S5. SHAP interaction analysis.

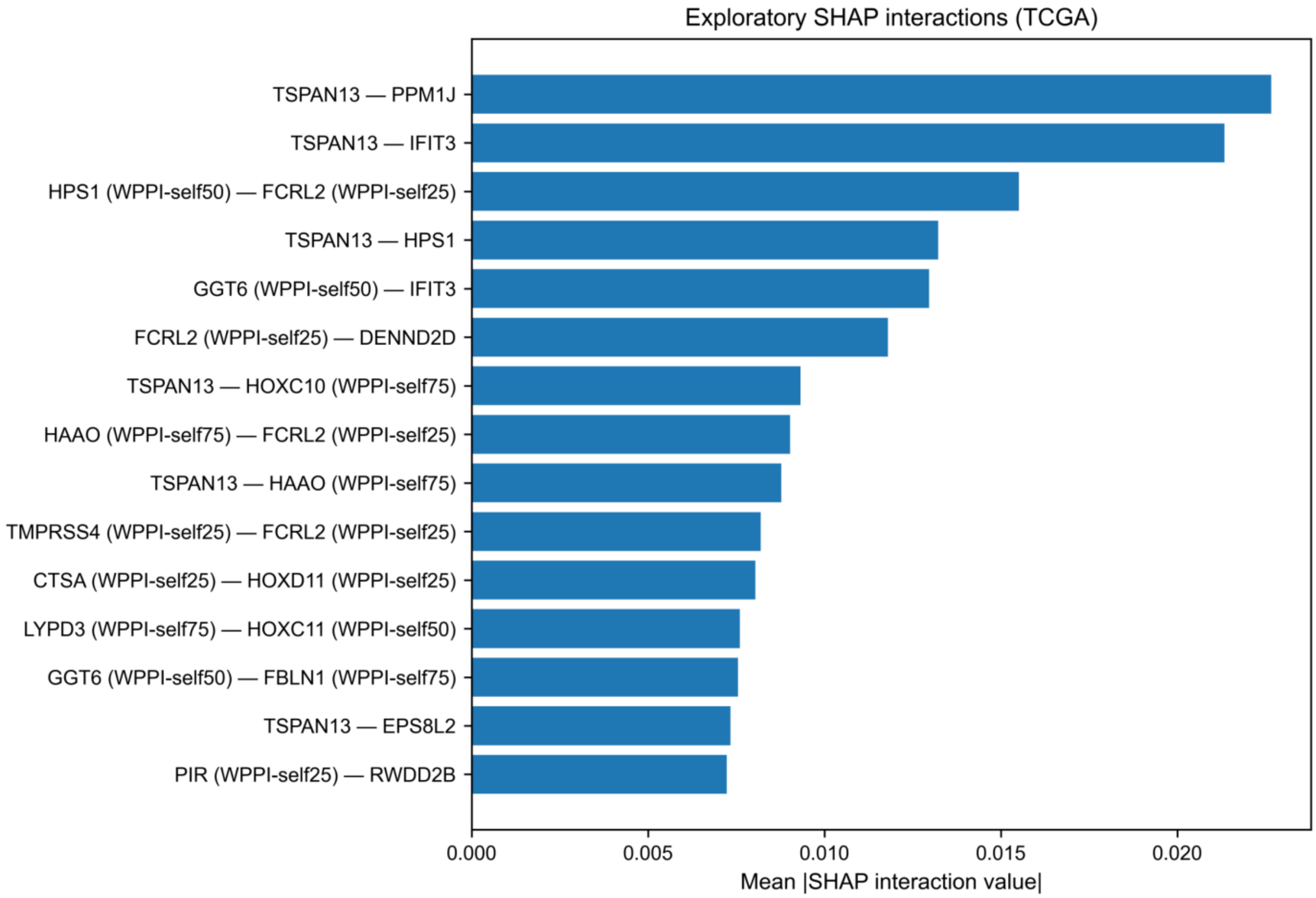

Legend. Exploratory post-lock SHAP interaction analysis of the frozen GIBD-XGBoost K100 model in TCGA. Interaction values represent model-level nonlinear dependency patterns and not biochemical interactions, PPI validation, or causal mechanisms.

Supplementary Figure S6. SHAP targeted dependence analysis.

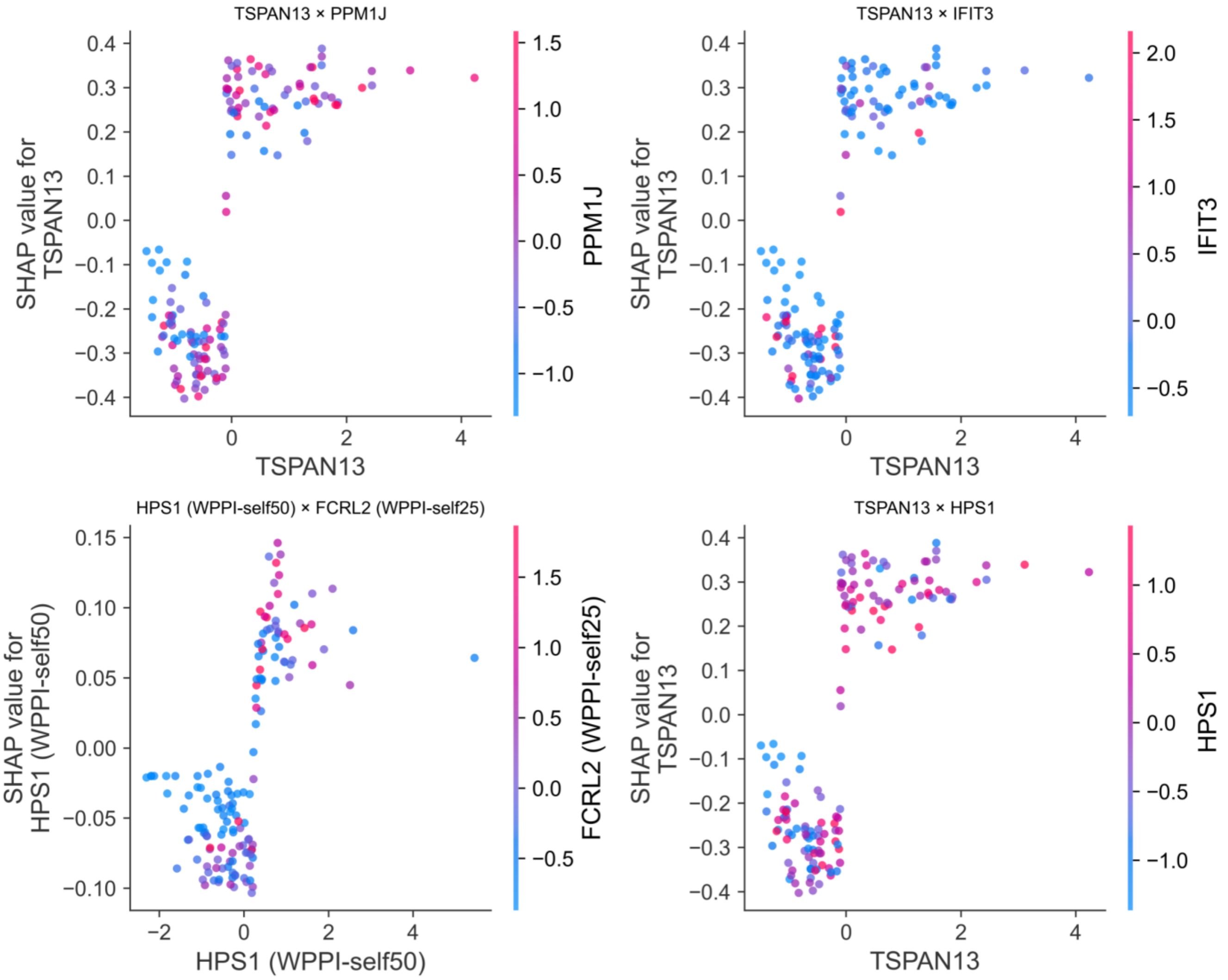

Legend. Exploratory targeted SHAP dependence analysis of selected influential feature interactions in the frozen GIBD-XGBoost K100 model. These plots were used for model-level interpretability only and did not influence feature selection, threshold selection, pathway analysis, or model fitting.

Supplementary Figure S7. Locked-signature ORA dotplots.

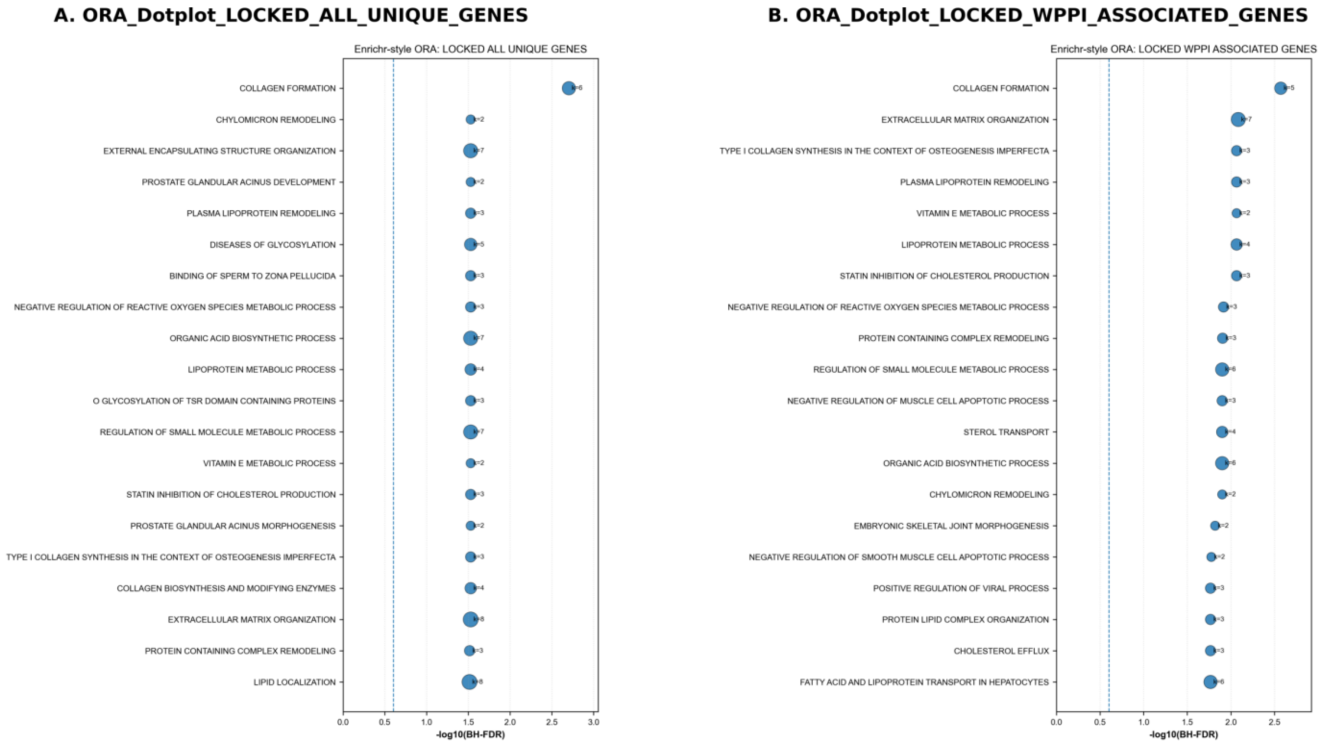

Legend. Locked-signature over-representation analysis dot plots for the final locked gene sets. Panel A shows ORA results for all unique locked genes, and Panel B shows ORA results for locked WPPI-associated genes. ORA was treated as supplementary pathway contextualization below full-transcriptome GSEA and was not used for feature selection, model tuning, threshold selection, or model validation.

Supplementary Figure S8. Supplementary Hallmark GSEA / extended GSEA outputs.

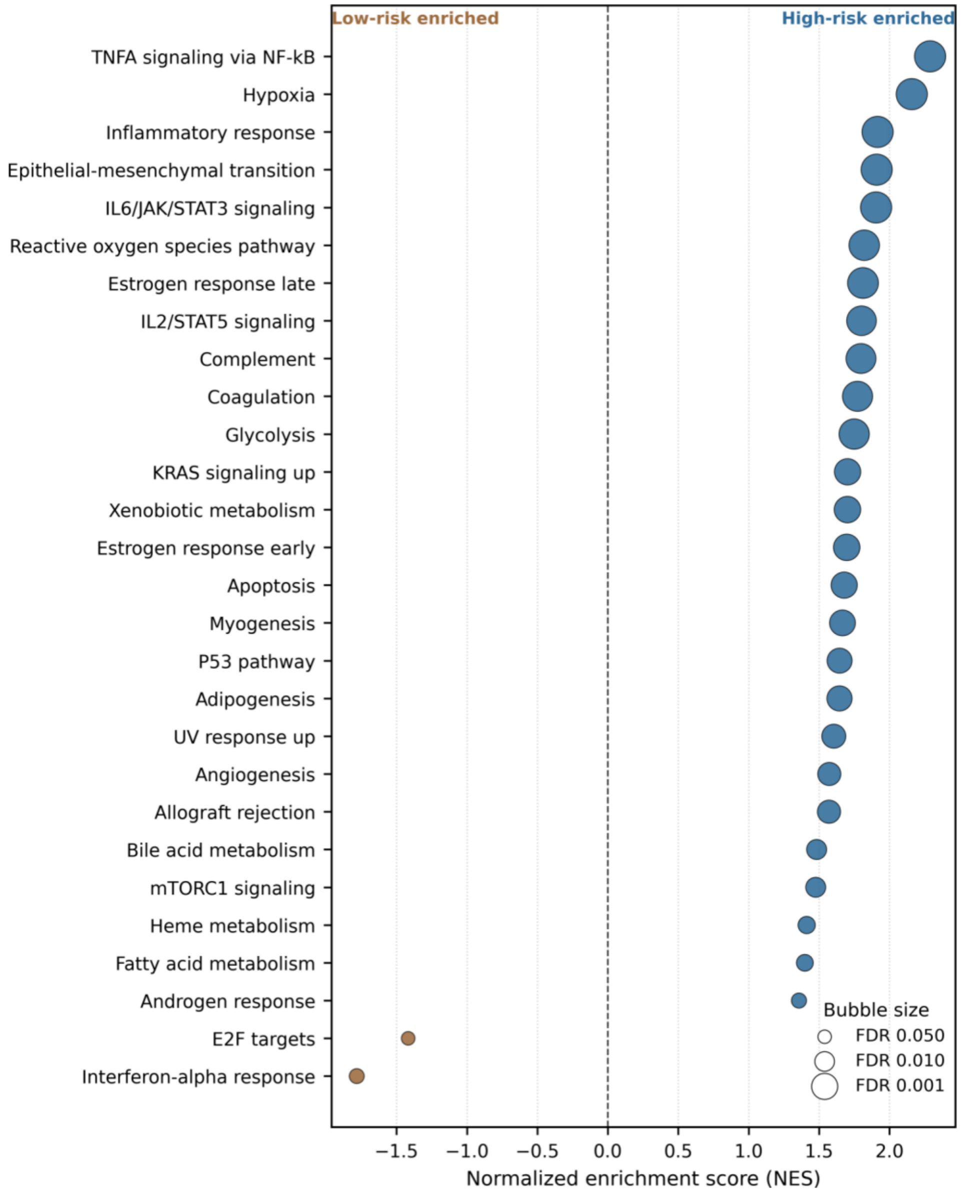

Legend. Extended Hallmark GSEA results for TCGA median-OS risk groups at FDR <0.05. Positive normalized enrichment scores indicate high-risk enrichment, whereas negative normalized enrichment scores indicate low-risk enrichment. This analysis was used for post-lock pathway contextualization and not for model development, feature selection, threshold selection, or validation.

Supplementary Tables

Supplementary Table S1. WPPI-self feature construction pseudocode.

Legend. Pseudocode for WPPI-self feature construction. STRING v12 topology was used as an external graph prior. Edges were retained using a combined-score cutoff of 700 or higher. For each target gene, measured non-self network neighbors were aggregated using confidence-weighted gene-level weights. Unmapped or unmeasured neighbors were excluded rather than zero-filled, and WPPI-self features were generated only when at least two measured non-self neighbors were available. Alpha values of 0.25, 0.50, and 0.75 were used to preserve target-gene expression while incorporating weighted neighbor context.

Step	Operation	Description
1	Input	Use log-CPM-normalized RNA-seq expression matrix and STRING v12 protein-protein interaction table.
2	STRING filtering	Retain STRING edges with combined score >=700 and scale edge confidence as combined_score / 1000.
3	Gene-protein mapping	Map measured genes to STRING protein identifiers; retain only genes present in the expression matrix.
4	Neighbor selection	For each target gene, collect valid measured non-self network-neighbor genes.
5	Missing-neighbor handling	Exclude unmapped or unmeasured neighbors rather than zero-filling them.
6	Minimum-neighbor rule	Generate WPPI-self features only when at least two valid measured non-self neighbors are available.
7	Weight aggregation	Accumulate confidence weights across retained protein-protein mappings and normalize gene-level neighbor weights.
8	Neighbor signal	Compute the weighted neighbor expression signal from measured neighbor-gene expression vectors.
9	Self-preserving mixture	Create WPPI_self_alpha = alpha * target_gene_expression + (1-alpha) * weighted_neighbor_signal.
10	Alpha values	Generate candidate features for alpha = 0.25, 0.50, and 0.75.
11	Output	Return WPPI-self candidate features for TCGA-only feature selection; do not generate pure neighbor-only features for the final locked space.

Supplementary Table S2. Reproducibility summary.

Legend Summary of reproducibility information for the locked median-OS K100 GIBD workflow, including final model configuration, stochastic seeds, script run order, major package versions, locked feature artifacts, and post-lock analysis outputs. Only major packages directly relevant to the final locked analysis are listed; standard-library modules and environment-only packages are omitted.

Item	Final locked value
Locked branch	Empirical median-OS K100
Final model	GIBD-XGBoost K100
Endpoint	TCGA empirical median OS = 357 days
Final feature count	100 features: 65 WPPI-derived and 35 raw-expression
Locked threshold	0.53
TCGA cohort	n=147; high-risk=74; low-risk=73
CGGA binary-evaluable cohort	n=131 from 133 eligible; high-risk=46; low-risk=85
Development/validation separation	TCGA only for development and locking; CGGA used post-lock for external validation metrics only
Thresholding rule	recall80_spec25
XGBoost hyperparameters	max_depth=3; learning_rate=0.029; n_estimators=85; gamma=0.18; reg_alpha=0.30; reg_lambda=6.5; scale_pos_weight=2.4; subsample=0.80; colsample_bytree=0.75; min_child_weight=1.8

Major package versions from the final analysis environment:

Package	Version
Python	3.13.5
pandas	2.3.3
numpy	2.3.4
scikit-learn	1.8.0
scikit-survival	0.27.0
xgboost	3.0.5
scipy	1.16.2
matplotlib	3.10.7
shap	0.49.1
lime	0.2.0.1
gseapy	1.1.13
joblib	1.5.2

Supplementary Table S3. PPI threshold sensitivity audit.

Legend. Post-lock PPI confidence-threshold sensitivity audit assessing graph coverage at STRING combined-score thresholds of 400, 700, and 900. The audit was descriptive and did not retrain models, reselect features, modify thresholds, use CGGA labels, or optimize CGGA performance.

STRING cutoff	Edges retained	Expression-eligible genes	Locked unique gene coverage	Locked WPPI-associated coverage	Interpretation
>=400	1,858,944	18,929/19,944 (94.9%)	90/90	58/58	Permissive graph; high coverage but higher low-confidence network density
>=700	473,860	14,216/19,944 (71.3%)	78/90	58/58	Selected high-confidence, coverage-preserving STRING cutoff
>=900	201,712	10,156/19,944 (50.9%)	54/90	40/58	Sparse graph; reduced locked WPPI-associated gene coverage
